# Stem cell-based therapy for fibrotic diseases: mechanisms and pathways

**DOI:** 10.1186/s13287-024-03782-5

**Published:** 2024-06-18

**Authors:** Marjan Taherian, Paria Bayati, Nazanin Mojtabavi

**Affiliations:** 1 Department of Immunology, School of Medicine, Iran University of Medical Sciences, Tehran, Iran; 2 Immunology Research Center, Institute of Immunology and Infectious Diseases, Iran University of Medical Sciences, Tehran, Iran

**Keywords:** Stem cell therapy, Fibrosis, Induced pluripotent stem cells, Mesenchymal stem cells, Coronavirus infections

## Abstract

Fibrosis is a pathological process, that could result in permanent scarring and impairment of the physiological function of the affected organ; this condition which is categorized under the term organ failure could affect various organs in different situations. The involvement of the major organs, such as the lungs, liver, kidney, heart, and skin, is associated with a high rate of morbidity and mortality across the world. Fibrotic disorders encompass a broad range of complications and could be traced to various illnesses and impairments; these could range from simple skin scars with beauty issues to severe rheumatologic or inflammatory disorders such as systemic sclerosis as well as idiopathic pulmonary fibrosis. Besides, the overactivation of immune responses during any inflammatory condition causing tissue damage could contribute to the pathogenic fibrotic events accompanying the healing response; for instance, the inflammation resulting from tissue engraftment could cause the formation of fibrotic scars in the grafted tissue, even in cases where the immune system deals with hard to clear infections, fibrotic scars could follow and cause severe adverse effects. A good example of such a complication is post-Covid19 lung fibrosis which could impair the life of the affected individuals with extensive lung involvement. However, effective therapies that halt or slow down the progression of fibrosis are missing in the current clinical settings. Considering the immunomodulatory and regenerative potential of distinct stem cell types, their application as an anti-fibrotic agent, capable of attenuating tissue fibrosis has been investigated by many researchers. Although the majority of the studies addressing the anti-fibrotic effects of stem cells indicated their potent capabilities, the underlying mechanisms, and pathways by which these cells could impact fibrotic processes remain poorly understood. Here, we first, review the properties of various stem cell types utilized so far as anti-fibrotic treatments and discuss the challenges and limitations associated with their applications in clinical settings; then, we will summarize the general and organ-specific mechanisms and pathways contributing to tissue fibrosis; finally, we will describe the mechanisms and pathways considered to be employed by distinct stem cell types for exerting anti-fibrotic events.

## Background

Fibrosis results from chronic organ injury and is typically characterized by tissue hardening and scarring caused by the excessive synthesis and deposition of disorganized extracellular matrix (ECM) components. Although ECM deposition is an inevitable and reversible part of normal wound healing, this process can become dysregulated if tissue irritant is severe enough or repetitive to sustain the production of pro-fibrotic factors, including cytokines, growth factors, angiogenic factors, and proteolytic enzymes. These factors contribute to the formation of excess fibrous connective tissue and progressive architectural remodeling that destroys organ structure [1–3]. Fibrotic disorders can eventually lead to death due to organ malfunction and failure, as seen in the end-stage of idiopathic pulmonary fibrosis (IPF), liver cirrhosis, cardiovascular disease, and progressive kidney disease [4–8]. Furthermore, fibrosis is implicated in tumorigenesis and tumor progression through excessive ECM accumulation that provokes cellular proliferation and alters cell polarity allowing cancer development and growth [9, 10].

The fundamental cellular mediators of fibroproliferative diseases are myofibroblasts with a particular contractile/synthetic phenotype, which is defined as strongly activated collagen-secreting, alpha-smooth muscle actin-positive (*α*-SMA^+^) fibroblasts. Myofibroblasts are responsible for excess production, remodeling, and contraction of ECM [2]. Myofibroblast differentiation can occur following tissue damage by multiple stimuli like various infections, chemical insults, autoimmune reactions, allergic responses, and mechanical injuries. The origin of myofibroblasts comprises resident fibroblasts, mesenchymal cells, and epithelial and endothelial cells in a trans-differentiation process known as epithelial to mesenchymal transition (EMT), and from circulating fibrocytes and bone-marrow-derived stem cells [11]. This process is normally limited to tissue healing. However, repetitive injuries and repair lead to uncontrolled myofibroblast activity and dysregulated ECM synthesis, and the eventual formation of a permanent fibrotic scar. Damaged epithelial and/or endothelial cells and matrix metalloproteinases (MMPs) produced by myofibroblasts, increase blood vessel permeability by disrupting the basal membrane, allowing macrophages, lymphocytes, and other immune cells to infiltrate [12]. Thereby, a chronic inflammatory environment is created, in which a large amount of pro-fibrotic cytokines and growth factors like transforming growth factor-beta (TGF-β), Wingless/Int-1 (Wnt1), IL-13, and platelet-derived growth factor are secreted (PDGF) [2, 13]. Several pathways such as TGF-β/ Smad2/3 and WNT/ CBP/β-catenin signal transduction, strongly are linked to the pathophysiology of fibrosis [14]. TGF-β, as the master regulator of myofibroblast differentiation in fibrosis, acts via a well-known canonical signaling pathway, in which binding of TGF-β to TGF-β receptor 1 (TGFR1, also known as ALK5) promotes downstream signaling that leads to phosphorylation and activation of Smad2/3 and eventually translocation of this complex to the nucleus associated with Smad4 [15]. TGF-β through TGFR1 further activates several non-Smad pathways (also described as non-canonical pathways) including MAP kinase pathways, phosphatidylinositol-3-kinase/AKT pathways, and Rho-like GTPase signaling pathways, which have been demonstrated to play role in fibrosis [16]. TGF-β1-induced transcription factors and WNT-stabilized β-catenin ultimately result in the expression of specific genes involved in further myofibroblast activation and production of ECM components such as collagen, fibronectin, and laminin. Moreover, yes-associated protein 1 (YAP)/transcriptional coactivator with PDZ-binding motif (TAZ) signaling, downstream of the Hippo signaling pathway, is involved in the expression of pro-fibrotic genes, such as connective tissue growth factor (CTGF) and PDGF that contribute in proliferation and activation of myofibroblasts through PI3K/AKT/mTOR pathway [14]. Targeting the fibrotic process in the involved organs remains a challenging prospect. Recently, the transplantation of various stem cell types has emerged as a promising therapeutic approach for fibrotic disease.

## Current stem cell applications in the field of fibrotic disorders

Stem cells (SCs) are undifferentiated precursor cells with two essential characteristics; First, unlimited self-renewal capacity, and second, the ability to give rise to various specialized cell types [17]. According to the last mentioned characteristic, stem cells are classified into two major categories; pluripotent which can differentiate into any cells in the adult body, and multipotent which differentiates into more limited cell types [18].

Pluripotent stem cells are primarily used in fibrotic therapy isolated from the inner cell mass of a blastocyst-stage embryo, hence named, embryonic stem cells (ESCs) [19, 20]. More recently, induced pluripotent stem cells (iPSCs), which are obtained by turning fully differentiated adult somatic cells back into an embryonic-like state [21–23], have been applied for the treatment of fibrosis [24, 25].

Multipotent stem cells are typically found in adult tissues or organs and have a restricted differentiation capacity depending on their location, where they aid in maintaining tissue integrity by replenishing the aging or damaged cells [26–28]. Mesenchymal stem cells (MSCs), also known as mesenchymal stromal cells, are multipotent SCs widely used to treat diverse fibrotic diseases [11]. Adult MSCs, for the first time harvested from bone marrow stroma via plastic adhesion, also can be isolated from other tissues, such as placenta, umbilical cord, amniotic fluid, adipose tissue, skeletal muscle, heart, lung, liver, kidney, Wharton’s jelly [29–31].

**Fig. 1:**
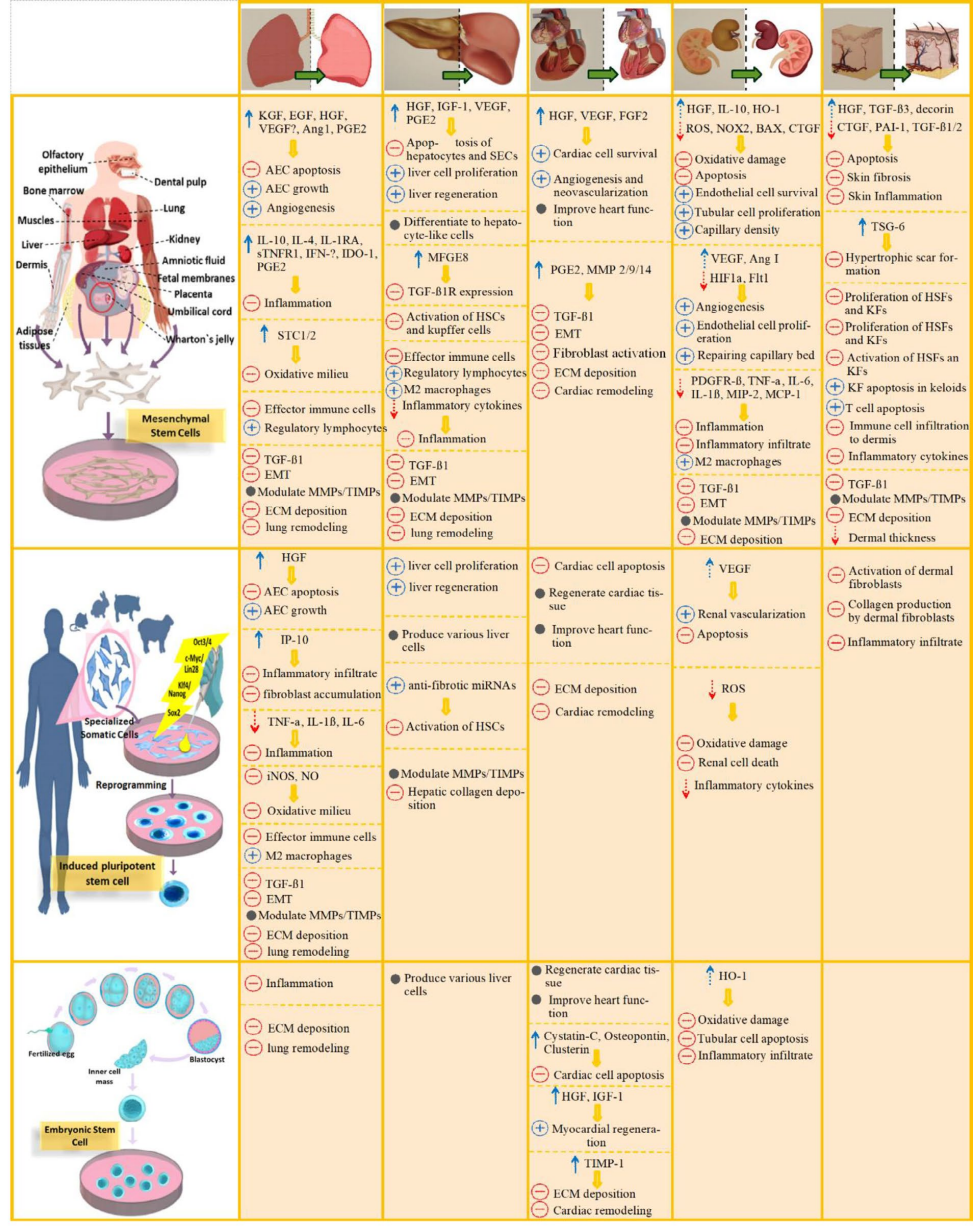
Schematic representation of preparing 3 distinct types of stem cells and the corresponding mechanisms exploited by each type to resolve various organ fibrosis in summary. Mesenchymal stem cells (MSCs), possible sources for isolating them, and their major anti-fibrotic effects are summarized in the first row. Induced pluripotent stem cells (iPSCs), and a brief schematic representation of their preparation basis, as well as their major anti-fibrotic functions, are summarized in the second row. Embryonic stem cells (ESCs), their in-vitro generation process, and their major anti-fibrotic effects are defined in the third row

Over the past decade, many efforts have been made to examine the applicability of stem cell-based therapy for diverse diseases that scarcely respond to available treatments. To date, MSCs, regardless of the originating sources, are the most widely stem cell type studied in stem cell-based therapy of fibrosis. MSCs exert immunomodulatory, anti-inflammatory, anti-proliferative, and anti-apoptotic properties. Despite the clinical application of MSCs for nearly ten years, about 75% of studies remained in phase II or earlier [32]. In fibrotic disorders, administration of ESCs or iPSCs is the promising approach to suppressing noxious pro-inflammatory and pro-fibrotic mechanisms and/or replacing the dysfunctional fibrotic tissues. In the following sections, we discuss the properties of various types of SCs and their advantages and challenges associated with every type of these SCs used in stem cell-based approaches. Fig. 1 schematically represents these cells and summarizes their application across different organ fibrosis as well as a brief description of their mechanism of action (Fig. 1).

### Mesenchymal stem cells

MSCs have become an attractive therapeutic option for treating chronic inflammatory disorders, autoimmune diseases, and fibrosis based on their ability in immunomodulation and anti-inflammatory characteristics. MSCs possess advantages, including ease of culture and availability, low immunogenicity, and fewer ethical debates [33]. Therefore, MSCs can be safely administered in either an autogenic or allogeneic manner to recipients due to a lack of host immune reactivity [34]. A wide range of clinical trials demonstrated that systemic administration of MSCs was well tolerated and not associated with significant short-term adverse events [35].

Despite these promising features, several concerns surrounding the efficiency and availability of MSCs due to their limited life span and undergoing senescence during in vitro expansion challenge their applicability [36]. The bulk of MSCs harvested from primary tissues is insufficient for any following application in clinical settings. Unlike ESCs and iPSCs, MSCs have a limited lifespan leading to significant changes in their phenotype and gene expression due to cell culture adaptation [37]. In addition, notable heterogeneity was demonstrated between separate subpopulations of MSCs when observed at the resolution of a single cell, even from a single source. Hence, another challenge involved a proper method to purify MSCs and ensure their homogeneity [38].

However, MSCs assumed to be a safer source than ESCs; their immunogenic and immunomodulatory properties need further elucidation. It is indicated that as opposed to mesenchymal progenitor cells separated from non-fibrotic lungs, the mesenchymal progenitor cell isolated from the lungs of IPF patients generate daughter cells that exert a transcriptional profile similar to that of IPF fibroblasts and fibrogenic activity to develop fibrotic lesions [39]. The other study by Waterman and colleagues challenged the widely accepted dogma that supposes MSCs are only immunosuppressive. This study revealed the polarization of two distinct phenotypes of MSCs following the involvement of specific TLRs. TLR3 activation led to upregulated fibronectin deposition, expression of immune-dampening mediators, and sustained T-cell inhibition. Conversely, TLR4 activation led to collagen deposition, expression of pro-inflammatory mediators, and reverse of the MSC-established suppressive mechanisms of T-cell activation [40]. These findings suggest that the immune-modulating activity of MSCs is more complex.

MSCs can be used in an allogeneic manner due to the low expression of MHC class I and II antigens allowing them to escape immune recognition. However, alloimmune response and immune rejection of allogeneic MSCs have been reported [41–43]. A few ischemic and non-ischemic heart failure patients have produced donor-specific antibodies against the MHC class I antigen, persisting for more than one month following the allogeneic MSC infusion. The expression of MHC antigens may be upregulated on the MSC surface in vivo [35]. Pro-inflammatory cytokines such as IFN-γ can upregulate the expression of MHC class I and II on MSCs [44, 45]. The finding can explain the cause of these conflicting results that MSCs enable to fluctuate the surface MHC class I and II profiles. A phenotype with high MHC class I and low or negative MHC class II expression was initially identified in MSCs from many species; however, MSCs from mice, humans, and horses with high levels of MHC class II were also described [41, 46, 47]. These findings collectively suggest that MSCs have a dynamic immune phenotype that can change their immune status.

#### Mesenchymal stem cells for the treatment of fibrotic diseases

After in vitro expansion and systemic administration by intravenous (IV) or intraperitoneal (IP) injection, MSCs tend to target sites of injury [48, 49]. Where they promote tissue repair, modulate immune responses, inhibit inflammation, and modify the microenvironment [48, 50]. MSCs also exert anti-apoptotic and anti-scarring properties favoring regression of fibrosis. MSCs mediate these effects directly or in a paracrine manner via secretome.

Since, MSCs exert different immunomodulatory capacities, proliferation properties, and therapeutic functions depending on their origin, we distinctly describe the anti-fibrotic properties associated with the MSCs derived from different sources.

#### Bone marrow-derived MSCs (BM-MSCs)

BM-MSCs were the first type of isolated MSCs and the vast majority of studies on stem cell-based therapy have examined their role in fibrosis, particularly fibrotic lung diseases [51]. BM-MSCs may have a more significant immunomodulatory potential than MSCs from other sources. BM-MSCs more effectively modulated the phenotypic transition of macrophages in several models of lung injury, compared with adipose-derived MSCs (AD-MSCs) [52, 53]. In co-culture with spleen mononuclear cells, BM-MSCs enabled suppression of the CD4 and CD8 expression, whereas AD-MSCs only suppressed the expression of CD4 [54]. A recent study showed that BM-MSCs displayed a higher immunomodulatory activity compared with AD-MSCs and Wharton’s jelly MSCs (WJ-MSCs). This study assessed the immunomodulatory activity based on the MSCs’ potency to inhibit the phytohemagglutinin-induced proliferation of peripheral blood mononuclear cells [55]. Collectively, BM-MSCs seem to be the best type of MSCs for immune-regulatory purposes.

However, there are several concerns regarding the administration of MSCs in some fibrotic diseases, such as idiopathic pulmonary fibrosis (IPF). The subpopulations of BM-MSCs are reported to have a fibrogenic nature and contribute to fibrosis progression. While delivered at the established fibrotic phase, they can acquire fibroblast or myofibroblast phenotype undergoing the local microenvironment in injured lungs [39, 49, 56]. According to this evidence supporting the potential improving effect of MSCs only by early intervention, MSC therapy for patients who have already developed pulmonary fibrosis is impractical. Recent studies found that although the administration of MSCs in the early stage during active inflammation might be more effective, fortunately, late administration of MSCs also has a therapeutic effect on established lung fibrosis [57]. By delayed injection, amniotic membrane MSCs (AM-MSCs), compared with BM-MSCs, were more effective in reducing inflammation and collagen deposition, and amelioration of established fibrosis in a repeated bleomycin (BLM) model of lung injury [58]. Thus, the therapeutic effect of MSCs impacted by the intervention time of administration.

As a therapeutic effect of MSCs is also impacted by donor-related factors such as allogeneic or autologous manner of transplantation, allogeneic BM-MSCs administration showed more efficacy in the treatment of lung injury than autologous BM-MSCs, which could be associated with the restricted auto-immunoregulatory capacity of autologous MSCs [59].

Although BM-MSCs exert great immunomodulatory properties, they showed a lower proliferation capacity and highest sensitivity to the stress microenvironment (oxygen and nutrient limitations) compared to AD-MSCs and WJ-MSCs. BM-MSCs exhibit a longer population doubling time (DT) and enter senescence after two passages. Whereas, the DT of WJ-MSCs is shorter than 24 h and stable for at least five passages [55]. Similarly, another study demonstrated the lowest proliferation capacity of BM-MSCs compared to that of AD-MSCs or umbilical cord MSCs (UC-MSCs) [60]. UC-MSCs showed a shorter DT than AD-MSCs [61], thus possessing the highest proliferation capacity among the MSCs mentioned above.

The in vivo therapeutic effects of BM-MSCs have been shown in various models and clinical trials. BM-MSCs augmented by granulocyte colony-stimulating factor (G-CSF) exert remarkable anti-fibrotic effects in animal models of lung injury [62]. Different studies have shown that the administration of BM-MSCs reverses the BLM-induced fibrotic effects; BM-MSCs play an influential role in improving lung fibrosis and ameliorating fibrosis symptoms [63–66]. However, extra-pulmonary alterations and senescence have been indicated in BM-MSCs from IPF patients, promoting inflammation and senescence in the local microenvironment [67]. A more recent study reported the clinical and functional progression in IPF patients who received an endobronchial infusion of BM-MSCs during a phase I clinical trial. This study also found some genomic instability in BM-MSCs cultured, which may be unfavorable using autologous MSCs [68].

The anti-fibrotic activity of BM-MSCs has also been shown in several investigations of renal fibrosis. BM-MSCs or their conditional medium mitigated disease in adenine, cisplatin, adriamycin-induced animal models, unilateral ureteral obstruction (UUO), and ischemia-reperfusion injury model [69–72]. Moreover, the ability of BM-MSCs to differentiate into hepatocyte-like cells (HLCs) in vitro [73] and liver restoration in hepatic failure have been shown [74]. However, the results from clinical trials regarding the therapeutic effect of BM-MSCs in improving histologic fibrosis remained controversial [75, 76].

#### Umbilical cord MSCs-derived (UC-MSCs) and placenta-derived MSCs (P-MSCs) and amnion-derived MSCs (AM-MSCs)

Some challenges regarding BM-MSCs, such as the low proliferation capacity, painfulness, and invasive isolation procedure, derived attention toward alternative sources. The alternative sources include the umbilical cord, amniotic membrane, and discarded test-tube human embryos, which are treated as biological waste and exhibit great proliferation activity, low immunogenicity, and high stem cell plasticity/phenotype [61, 77]. UC-MSCs were indicated to preserve proliferation capacity for greater than 90 population doublings without senescence while maintaining MSC properties and functions [78]. Numerous studies have addressed the safety, anti-inflammatory, and anti-fibrotic activity in different diseases with inflammatory and fibrotic etiology, including lung fibrosis [38, 58, 79, 80], liver fibrosis [81–83], heart failure [84, 85] and COVID-19 [86, 87]. The immunosuppressive functions of UC-MSCs have been reported to mediate by recruiting regulatory T cells, via their interaction with macrophages during the repair process of BLM-induced lung fibrosis [38]. The in vivo anti-fibrotic activity of UC-MSCs has also been linked to the downregulation of the IL-6/IL-10/TGFβ axis involving lung M2 macrophages [79]. Human UC-MSCs and their exosomes could attenuate liver fibrosis induced by CCl4 in mice [81, 82]. UC-MSC transplantation showed to be effective in both regression of liver fibrosis and reducing related ascites in patients [83]. Because of these potent immunomodulatory and anti-inflammatory effects, UC-MSCs have been recently suggested to be useful for dampening the excessive inflammatory response in the lungs, leading to acute lung injury, acute respiratory distress syndrome (ARDS), organ failure, and death in the severe COVID-19 patients [86–88].

Compared with ESCs, UC-MSCs are less readily available, whereas P-MSCs can engraft in solid organs after xenotransplantation [89]. The administration of P-MSCs effectively mitigated BLM-induced lung fibrosis along with the inhibition of neutrophil infiltration [90], and suppression of pro-fibrotic cytokines [91]. P-MSC infusion was feasible and safe in IPF patients and associated only with stable disease function and severity [92].

AM-MSC transplantation reduced inflammation and alleviated BLM-induced lung fibrosis in mice [93]. Extracellular vesicles derived from AM-MSCs ameliorated hepatic inflammation and fibrogenesis [94], oxidative stress, inflammatory cytokines, TGF-β, and α-SMA, as well as improving the microvascular dysfunction and portal hypertension in the CCl4-induced liver fibrosis rat model [95].

#### Adipose tissue-derived mesenchymal stem cells (AD-MSCs)

AD-MSCs are considered an acceptable alternative for BM-MSCs because of their advantages, including ease of isolation via liposuction with minimal discomfort to patients, more abundance, potentially higher stemness, and more in vitro proliferation and expansion capacity without entering senescence, producing a higher amount of bioactive mediators such as hepatocyte growth factor (HGF) and cytokine (IL-1, IL-6, IL-8) receptor antagonists [96–98].

A large body of evidence showed the anti-fibrotic efficacy of AD-MSCs in the improvement of lung fibrosis [99–102], liver fibrosis [103, 104], renal fibrosis [98], and dermal fibrosis [105–107]. Chen and colleagues indicated that the AD-MSCs-mediated anti-pulmonary fibrosis effect involved the anti-inflammatory and anti-apoptosis activities, which are promoted by reducing the pulmonary inflammatory response (downregulation of TNF-α, IL-1β, IL-6, and IL-10) and inhibition of mitochondrial apoptosis-related protein (Caspase-3) expression. Thereby, diminished pulmonary fibrosis of silicosis in rats [102]. Consistently, another study demonstrated the therapeutic effect of AD-MSCs in both inflammatory and fibrotic phases of BLM-induced interstitial lung disease in mice. AD-MSCs achieved that by inhibiting pro-inflammatory cytokines (TNF-α and IL-12) in activated macrophages, inducing the apoptosis of activated macrophages, suppressing the differentiation/proliferation of Th2 cells, and promoting the differentiation/proliferation of regulatory T cells [100]. Although the vast majority of studies comply with these, the controversial findings obtained by Uji and colleagues demonstrated that intravenous injection of AD-MSCs was inefficient for the amelioration of BLM-induced lung injury in rats [108]. In another study, they further examined the intratracheal route of administration and showed that AD-MSCs did not affect the severity of lung damage at the onset of disease, but prevented the ongoing aggravation of lung injury in the long term [109]. Although the intravenous administration of AD-MSCs and their lack of homing capacity are suggested as the probable reason for observed treatment failure, previous research provided evidence of the protective effect of intravenously administrated AD-MSCs against BLM-induced lung fibrosis, particularly in early-stage [100, 101, 110]. The older animal’s age and stage of the fibrotic disease are likely the other reasons. Notably, the anti-fibrotic activity of AD-MSCs was shown to be age-dependent, as young-donor-derived AD-MSCs, in contrast to old-donor-derived AD-MSCs, inhibit fibrosis in the aged animal [110].

Moreover, the intravenously administrated autologous AD-MSCs in COVID-19 patients were evaluated for safety and prophylactic efficacy in a phase II study and received FDA approval [111]. The comparative study on BM-MSCs and AD-MSCs in the treatment of rat model of CCl4-induced liver fibrosis indicated that although both of them are similarly effective at attenuating liver fibrosis by promoting the apoptosis and suppressing the activation and proliferation of hepatic stellate cells (HSCs), AD-MSCs were relatively more effective in anti-inflammatory and anti-liver fibrotic activities [103]. Injected AD-MSCs into UUO model rats via tail vein, or intraperitoneally into ischemia-reperfusion injury (IRI) mice resulted to reduce EMT, α-SMA, fibroblast-specific protein 1 (FSP-1), and ameliorate inflammatory response and renal interstitial fibrosis [112, 113].

Taken together, the anti-fibrotic efficacy of MSCs affected by; (i) source-related factors, including immunomodulatory potency, stemness characteristics, the potential of multi-linage differentiation, proliferation properties, n, (ii) donor-related factors, including immunogenicity (allogeneic or autogenic transplantation), age, sex and health status (such as obesity) [114, 115], (iii) intervention related parameters, such as time, infusion manner, dose, and experimental model. Considering these variables, definitive comparisons between investigations are obscured, although more frequent of them endorsed that MSCs can attenuate fibrosis.​.

### Embryonic stem cells

To date, ESCs used to cure different degenerative and/or inflammatory diseases. Theoretically, the pluripotent nature of ESC makes them an ideal candidate to regenerate and replenish damaged tissues [116]. In the case of fibrosis, cell populations derived from ESCs have been shown to display immunomodulatory, anti-inflammatory, and anti-fibrotic functions.

However, several impediments make ESC-based therapy a challenging effort. Since the isolation of ESCs leads to the destruction of embryos, many ethical and legal obstacles restrict the clinical application of these cells. Moreover, their strong proliferative potential and multi-lineage differentiation capacity may result in teratoma formation when ESCs injected in vivo before commitment [117]. ESCs present foreign antigens and immune rejection can occur following transplantation [118]. Propagation of ESCs, preserving their undifferentiated state, and differentiation into desirable cell lines in cell culture are complex technical challenges [119].

#### Embryonic stem cells for the treatment of fibrotic diseases

Intramyocardial injection of mouse ESCs inhibits cardiac fibrosis in the infarcted heart of C57BL/6 mice [120]. Pneumocytes derived from in vitro differentiation of ESCs reduced inflammation and fibrosis markers and recovered lung injury in the pulmonary fibrosis model [121]. Furthermore, amelioration of pulmonary fibrosis was observed after the transplantation of differentiated human ESCs (into lung epithelial lineage-specific cells) in the bleomycin mouse model of IPF. Interestingly, amelioration of lung injury was also revealed in regions that did not harbor engrafted cells, suggesting that differentiated human ESCs promoted anti-fibrotic effects via direct and indirect (paracrine) mechanisms [122]. In a recent study, Wu and colleagues reported the generation of clinical-grade human embryonic stem cells (hESCs)-derived immunity- and matrix-regulatory cells (IMRCs), which avoid the ethical controversy of ESCs and heterogeneity among subpopulations of MSCs. IMRCs mimicked the MSCs in their ability of self-renewal and multi-lineage differentiation and exhibited a higher immunomodulatory capacity and anti-fibrotic activity compared with UC-MSCs. In addition, they were superior to UC-MSCs and pirfenidone in treating lung injury and fibrosis, with excellent efficacy and safety profiles in mice and monkeys [123]. In a study by Liu et al. (2023) they administered human embryonic stem cell exosomes (hESC-exo) to bleomycin-induced mouse model of IPF from the first day after treatment. Their findings revealed that hESC-exo notably alleviated inflammation, removed collagen deposition, and restored alveolar architecture in the lungs. They have further shown that miR-17-5p within hESC-exo directly targeted thrombospondin-2 (Thbs2), which modulates inflammation and fibrosis, thereby protecting against bleomycin-induced lung toxicity through the miR-17-5p/Thbs2 axis [124].

### Induced pluripotent stem cells (iPSCs)

iPSCs are promising candidates superior to the pre-existing stem cells for regenerative therapy. iPSCs generated by reprogramming somatic cells with ectopic expression of specific pluripotency genes to acquire self-renewal ability and the potential for differentiation into all cell types of the body [21–23]. iPSCs; first, closely resemble ESCs, thus providing the opportunity to bypass potential issues of allogeneic immune rejection and ethical concerns about isolation and use of human ESCs [125]; second, possess the capacity of unlimited replication to produce quasi-identical genetic and functional properties, thus can bypass several concerns surrounding proliferation characteristics, different genetic background, and heterogeneity of MSCs [126].

Furthermore, iPSCs can be generated directly from patient-specific somatic cells and transplanted in an autologous manner despite ESCs [127]. Derivative cells from iPSCs are unlikely to cause immune rejection upon transplantation [128]. Like ESCs, iPSCs present low or absent levels of MHC class I and are negative for MHC class II. Unlike MSCs, the expression of MHC class II on iPSCs is not upregulated during differentiation and IFN-γ stimulation. The results obtained thus far regarding the alteration in MHC class I expression upon differentiation or stimulation with pro-inflammatory cytokines, and immunogenicity of iPSCs are conflicting [129–131], presumably, due to a variety of reprogramming methods [132]. More notably, iPSCs have been found to possess more potent immunomodulatory effects in vitro, than BM-MSCs [133]. These characteristics offer iPSCs as an ideal candidate for anti-fibrotic therapy.

The pluripotency genes used for the generation of iPSCs include four reprogramming factors; Oct3/4 and Sox2 with either Klf4 and c-Myc or Lin28 and Nanog, which were initially introduced into the mouse and human somatic cell by viral transfection system (retroviruses, lentiviruses or adenoviruses) [21–23, 134]. However, neither the expression of oncogenic transcription factor c-Myc nor the viral delivery method is likely to be approved for human therapy. Subsequently, c-Myc-free iPSCs were generated using only three reprogramming genes, Oct-4/Sox2/Klf4, excluding c-Myc, to reduce tumorigenicity [135]. An integrated viral genome could raise the incidence of mutations and subsequent tumor formation after iPSC grafts. To resolve this problem, integration-free vectors have been designed, including expression plasmids [136], episomal plasmids [137], and Sendai virus-based vectors [138]. The generation of c-Myc- virus-free iPSCs, addresses a critical safety concern for the potential use of iPSCs for clinical application. To increase the transfection efficacy and safety, several approaches for generating the human iPSC free of reprogramming factors have also been developed, including Cre-recombinase excisable viruses [139], protein- [140], and mRNA-based methods [141].

Despite these advances in iPSCs technology, genomic instability and emerging genetic variations have remained a safety concern regarding tumorigenicity. Pre-existing variations in parental somatic cells, reprogramming-induced mutations during the reprogramming process, and passage-induced mutations during the prolonged culture, have been considered the major origins of genetic variations of iPSCs. Several variables of generation methods including the source of somatic cells, delivery method, reprogramming factors, and cell passage can affect genomic instability [142]. Towards clinical applications, it is essential to modulate these variables properly to produce iPSCs more efficiently and safely.

Further genomic, epigenomic, and functional assessment of the iPSCs produced by these new methods is crucial to understand whether there is an appropriate method that may allow a safer clinical application of iPSCs [143].

#### iPSCs for the treatment of fibrotic diseases

To date, most endeavors in cell-based therapy for organ-specific disorders have focused on two main areas; to make unlimited numbers of patient-specific tissue cells to regenerate the damaged organ, or to provide autologous genetically corrected cells for permanent corrective therapy of incurable and hereditary diseases of the liver [144], heart [145], kidney [146, 147], and skin [148–150]. Furthermore, some studies utilized a limitless cell supply obtained from patient somatic cell-derived iPSCs for iPSC-based disease modeling, which is used to examine pathologic mechanisms and pharmacological interventions in various diseases such as organ fibrosis and failure [151–155]. The studies in all the above-mentioned scopes have been excluded from this review.

Fortunately, intravenous administration of mouse c-Myc-free iPSCs, as well as their conditioned medium, has been indicated to attenuate BLM-induced pulmonary fibrosis. The protective mechanism includes the early amelioration of inflammation, reduced pro-inflammatory and pro-fibrotic cytokines and chemokine, and increased production of anti-fibrotic chemokine interferon-γ-induced protein 10 (IP-10) in the injured lungs. In addition, tumorigenesis was not detected within the 2-month follow-up after the transplantation of c-Myc-free iPSCs [24]. Similarly, another study confirmed the inhibitory effects of these cells in BLM-induced lung injury, via the reduction in lung wet/dry weight ratio, collagen deposition, body weight loss, and inflammatory mediators [156]. In the acute hepatic failure (AHF) murine model, intravenously transplanted mouse c-Myc-free iPSCs or iPSC-derived hepatocytes (iPSC-Heps) tended to migrate into the injured liver area, where they improved liver functions and rescued animals from lethal AHF. Notably, no tumor formation was reported in the c-Myc-free iPSC and c-Myc-free iPSC-Hep grafts six months after transplantation, despite of iPSCs with c-Myc [135]. Consistently, Caldas and colleagues reported that rat c-Myc-free iPSCs can retard chronic kidney disease (CKD) progression, but also develop Wilms’ tumors in rats [157]. So, they blocked the proliferative capacity of human iPSCs with mitomycin C and also differentiated human iPSCs into renal progenitor cells (RPCs) prior to the infusion to avoid tumor formation in their next study. They observed the beneficial effects of both cell types in attenuating CKD progression, which was indicated by improvement of clinical and histological CKD parameters, including decreased tubulointerstitial damage (interstitial fibrosis and tubular atrophy), glomerulosclerosis and α-SMA. However, human iPSCs, compared with RPCs, were shown to be more efficient, presumably as a consequence of their paracrine effect [158]. According to their evidence, tumor formation promoted by the iPSCs seems to remain a limitation, although the technology allowing the production of c-Myc-free iPSCs avoids oncogenesis. Thus, this observation has been linked to genomic instability and the incidence of mutation [158]. However, it is not clear that these mutations could lead to adverse events. Further investigations are required to characterize genetic variations and to find which mutations in iPSCs can confer adverse effects such as malignant outgrowth.

Although these investigations underlying the use of iPSCs in organ fibrosis showed promising results, there are only a few publications regarding the anti-fibrotic function and possible mechanism of iPSCs, when administered before differentiation. Other studies instead examined the effect of iPSCs-derived cell lineages or the iPSCs-conditioned medium on fibrosis.

#### iPSC-derivative cell lines for the treatment of fibrotic diseases

A new approach to exploring the applications of iPSCs in organ fibrosis is using derivative cell lines obtained by in vitro differentiation of mouse or human iPSCs. Derivation of endoderm and then distal alveolar epithelial type II cells (AEC2)-like, alveolar epithelial type I cells (AEC1)-like, and proximal lung cells from iPSCs have been well characterized in vitro [159–161], and promising results with the transplantation of iPSC-derived epithelial cell progenitors reported in vivo.

The intratracheal administration of mouse iPSC-derived AEC2 inhibited lung inflammation and collagen deposition, and abrogate lung injury in the BLM-induced mouse acute lung injury model [162]. Another study recently showed that intratracheal transplantation of human iPSC-AEC2 in the rat model of BLM-induced lung fibrosis 15 days after BLM challenge, was able to reduce disease severity, when fibrosis has already developed, by suppressing both TGF-β and α-SMA expression and decreasing the collagen deposition [163]. Importantly, it was the first report of the effectiveness of this approach during the fibrotic stage of the disease, when fibrosis had been fully developed. Hence, address the issue regarding the feasibility of iPSC-based therapy in patients who already developed fibrosis.

Consistently, intravenously injected human iPSC-derived lung epithelial cell progenitors (LECs) enriched by magnetic-activated cell sorting (MACS) for CD166 (a selective marker for early lung progenitor cell) integrate into the lung alveoli of BLM-injured NOD/SCID mice, increased the survivability of mice, reduced the lung damage and reactive fibrosis and improved pulmonary function. However, these protective effects of iPSC-LECs supposedly were less signified in the survival rates, compared to those observed with LECs derived from hESs [164]. The cause of this observation has been linked to epigenetic profiles, as a principal difference between ESCs and iPSCs described by several studies. iPSCs exert the epigenetic memory and retain the transcriptional memory of the original cells [165, 166]. Moreover, iPSCs express a unique signature, which could be related to ineffective silencing of the gene expression pattern of original cells [167]. The inherent genetic and epigenetic hallmarks of iPSCs may lead to less efficient diffraction into various somatic cell types aside from their originating cell type [164]. This information emphasizes the significance of comprehensive profiling of iPSC lines to determine those relevant for a convenient and safe application.

#### iPSC secretome for the treatment of fibrotic diseases

Because of its acellular nature, the use of iPSCs-conditioned medium and secretome, containing the paracrine-secreted products, is considered an alternative to circumvent the safety concerns and long-term effects of iPSCs [168]. The intratracheally instilled human iPSCs-conditioned medium was shown to reverse fibrosis in the bleomycin-injured rat lungs [169]. This anti-pulmonary fibrosis of intratracheal iPSCs-conditioned medium was subsequently reported to be partially mediated by hepatocyte growth factor (HGF), accompanied by reduction of the collagen deposition, TGFβ1, and α-SMA expression in rat lungs [170]. Another report confirmed the protective effects of the iPSCs-conditioned medium on BLM-induced lung fibrosis in mice, in part through the TGF-β1-related pathway [171]. Further investigation to indicate the anti-fibrotic mechanism of iPSC secretome highlighted the alteration in phenotype and gene expression pattern of interstitial macrophages. The iPSCs-conditioned medium reduced fibrosis and the total number of macrophages (M1 and M2 phenotypes) in the BLM injured rat lungs, and microarray data showed involvement of three essential pathways; (a) branching morphogenesis, (b) immune regulation, and (c) tissue regeneration after injury [172]. In a more recent report, the anti-fibrotic and regenerative potential of the iPSCs-conditioned medium have been related to Amyloid precursor protein (APP) and ELAV-like protein 1 (ELAVL-1) as essential components of the iPSC secretome that that contributes to change the secretory pattern and gene expression of macrophages towards anti-fibrotic phenotypes in vitro [173].

Moreover, the anti-fibrotic effect of human iPSC- extracellular vesicles (EVs) showed at protein and gene levels to reverse liver fibrosis in two murine models of liver injury by CCl4 and bile duct ligation. These EVs enabled the reduction of pro-fibrogenic markers such as α-SMA, CollagenIα1, and fibronectin in the hepatic stellate cell (HSC) and hepatic collagen deposition [174].

In the following highlights, the major points in utilizing each type of stem cell for antifibrotic purposes are summarized:

#### Mesenchymal stem cells (MSCs)

- MSCs are a promising cell-based therapy for chronic diseases due to their immunomodulatory and anti-inflammatory properties.
- They can be obtained from various sources like bone marrow, adipose tissue, and umbilical cord.
- MSCs have limitations like low proliferation capacity and potential for becoming fibrotic themselves.
- Studies show conflicting results on the effectiveness of MSCs depending on the source, timing of administration, and disease model.

#### Mesenchymal stem cells for the treatment of fibrotic diseases

- MSCs target injured sites and promote tissue repair, modulate immune responses, and inhibit inflammation.
- Bone marrow-derived MSCs (BM-MSCs) have been most studied for treating fibrotic lung diseases.
- BM-MSCs may have a stronger immunomodulatory effect than MSCs from other sources.
- However, BM-MSCs from IPF patients may worsen fibrosis and allogeneic BM-MSCs might be more effective than autologous ones.
- Adipose tissue-derived MSCs (AD-MSCs) are an alternative to BM-MSCs due to easier isolation and higher proliferation capacity.
- Studies show the effectiveness of AD-MSCs in improving lung, liver, and kidney fibrosis.
- Umbilical cord MSCs (UC-MSCs) and Placenta-derived MSCs (P-MSCs) are gaining attention due to their ease of availability and immunomodulatory properties.
- Studies suggest their effectiveness in various fibrotic diseases, including lung fibrosis and COVID-19.

#### Embryonic stem cells (ESCs)

- ESCs are attractive for treating fibrosis due to their ability to differentiate into various cell types and their immunomodulatory properties.
- However, ethical concerns regarding embryo destruction and safety risks limit their clinical application.
- ESCs can form tumors (teratomas) if not properly managed before transplantation.
- The body may reject transplanted ESCs due to their foreign antigens.
- Maintaining ESCs in an undifferentiated state and differentiating them into desired cell lines is challenging.

#### ESCs for treating fibrotic diseases

- Studies show that ESC-derived cells can reduce inflammation and fibrosis in animal models of lung disease.
- Transplanted ESCs can improve lung function through direct and indirect (paracrine) mechanisms.
- Recent studies have developed human ESC-derived immunity- and matrix-regulatory cells (IMRCs) that avoid ethical concerns and show promise in treating lung fibrosis.
- ESC-derived exosomes containing microRNAs may also be a therapeutic approach for fibrosis.

#### Induced pluripotent stem cells (iPSCs)

- iPSCs are reprogrammed adult cells that hold promise for regenerative medicine due to their pluripotency and ability to be patient-specific, avoiding immune rejection.
- Safety concerns exist due to the risk of tumor formation from residual reprogramming factors and genetic variations.
- c-Myc-free iPSCs and integration-free vectors for reprogramming are being developed to improve safety.

#### iPSCs for treating fibrotic diseases

- Studies using c-Myc-free iPSCs show promising results in reducing fibrosis in animal models of lung and liver diseases.
- iPSCs can be used for disease modeling to understand the mechanisms of fibrosis.
- Tumor formation remains a concern, although c-Myc-free iPSCs mitigate this risk.
- Further research is needed to understand the long-term effects of iPSCs.

#### iPSC-derived cell lines for treating fibrosis

- Transplantation of iPSC-derived epithelial cells shows promise in treating lung fibrosis in animal models.
- iPSC-derived cells may be less efficient than ESC-derived cells due to epigenetic differences.
- Careful selection and profiling of iPSC lines is crucial for optimal therapeutic effects.

#### iPSC secretome for treating fibrotic diseases

- iPSC-conditioned medium containing paracrine factors offers a safer alternative to cell therapy for fibrosis.
- Studies suggest that iPSC secretome promotes an anti-fibrotic phenotype in macrophages and reduces fibrosis.
- Specific components of the iPSC secretome, like Amyloid precursor protein (APP) and ELAV-like protein 1 (ELAVL-1), may be key players in its anti-fibrotic effects.
- iPSC-derived extracellular vesicles (EVs) have also shown promise in reversing liver fibrosis.

Overall, MSCs currently appear to be the most promising option for treating fibrosis due to their safety, feasibility, and anti-fibrotic properties. However, research on iPSCs and ESCs is ongoing, and they hold future potential with advancements in technology and overcoming ethical concerns. Also, it should be noted that the optimal stem cell type for treating fibrosis might vary depending on the specific organ affected and the severity of the condition. Further research is needed to determine the most effective delivery methods and dosing regimens for each type of stem cell therapy as well. Table 1 summarizes the advantages and disadvantages of using each stem cell type as well as their application in ongoing clinical studies.

**Table 1:** Comparison of Stem Cell Types for Fibrosis Treatment. This table compares three major stem cell types (MSCs, iPSCs, ESCs) for their potential in treating fibrosis. It highlights their advantages, disadvantages, and ongoing clinical trial focuses

Stem Cell Type	Pros	Cons	Ongoing Clinical Trials (Focus)
MSCs (Mesenchymal Stem Cells)	**Immunomodulatory**: Suppress inflammation, a key driver of fibrosis. **Paracrine effects**: Secrete factors promoting tissue repair, inhibiting fibroblast activity, and stimulating angiogenesis. **Feasibility**: Readily isolated and expanded from adult sources (bone marrow, adipose tissue). **Safety**: Good safety profile with minimal risk of rejection due to low immunogenicity.	**Variability**: Effectiveness may vary depending on source, isolation method, and expansion procedures. **Mechanism**: Exact mechanisms of combating fibrosis are still being explored, making it challenging to optimize therapy. **Limited differentiation**: Promote tissue repair but have limited ability to directly differentiate into mature organ-specific cells.	There are numerous ongoing clinical trials using MSCs for various types of fibrosis. Examples include: Idiopathic Pulmonary Fibrosis (IPF), Liver Fibrosis, Cardiac Fibrosis (post-myocardial infarction), Kidney Fibrosis
iPSCs (Induced Pluripotent Stem Cells)	**Immunomodulatory**: Suppress inflammation, a key driver of fibrosis. **Paracrine effects**: Secrete factors promoting tissue repair, inhibiting fibroblast activity, and stimulating angiogenesis. **Versatility**: Theoretically differentiate into any cell type, offering the potential to replace damaged cells and directly address fibrosis. **Patient-specific cells**: Can be derived from a patient’s own cells, reducing rejection risk.	**Tumorigenesis**: Risk of uncontrolled cell growth and tumor formation if not fully differentiated or reprogrammed properly. **Technical challenges**: Technology for generating and manipulating iPSCs is still under development and expensive.	Clinical trials using iPSCs for fibrosis are less common than MSCs due to technical challenges. However, some early-stage trials are exploring their potential for treating Liver and Kidney Fibrosis
ESCs (Embryonic Stem Cells)	**Pluripotency**: Can differentiate into any cell type, offering high potential for tissue repair and regeneration.	**Ethical concerns**: Obtaining ESCs involves destroying a blastocyst (early-stage embryo), raising ethical concerns. **Immune rejection**: ESCs derived from another person are highly immunogenic, increasing rejection risk. **Limited availability**: Strict regulations surrounding ESC use limit their availability for research and therapy.	Owing to ethical considerations and practical constraints, the use of embryonic stem cells (ESCs) in current clinical trials targeting fibrosis remains limited. However, their secretory byproducts, particularly exosomes, have been investigated in preclinical research settings

## Pathogenic insights of organ fibrosis affected by stem cells

Fibrosis can occur in almost all tissues and organs in the body. Extensive tissue remodeling and aberrant wound healing in some diseases, such as systemic sclerosis, idiopathic pulmonary fibrosis, liver cirrhosis, cardiovascular fibrosis, and chronic kidney disease (CKD) cause a devastating fibrotic process, which can lead to organ failure and death. Here, we describe the pathogenesis underlying organ-specific fibrosis, with a focus on pathological mechanisms affected by stem cell therapy.

### Pulmonary fibrosis (PF)

The fibrotic process is shared with numerous lung diseases, including sarcoidosis, hypersensitivity pneumonitis, and pneumoconiosis. It is also observed as an adverse effect of some drugs. Pulmonary fibrosis is further accompanied by systemic inflammatory and autoimmune diseases or connective tissue disorders such as rheumatoid arthritis and systemic sclerosis [25]. This complication can also be secondary to lung infection, as a current example of COVID-19. A more frequent and progressive form of pulmonary fibrosis with unknown etiology and poor prognosis is idiopathic pulmonary fibrosis (IPF), which is also considered the most common type of interstitial lung disease (ILD) [175].

**Fig. 2:**
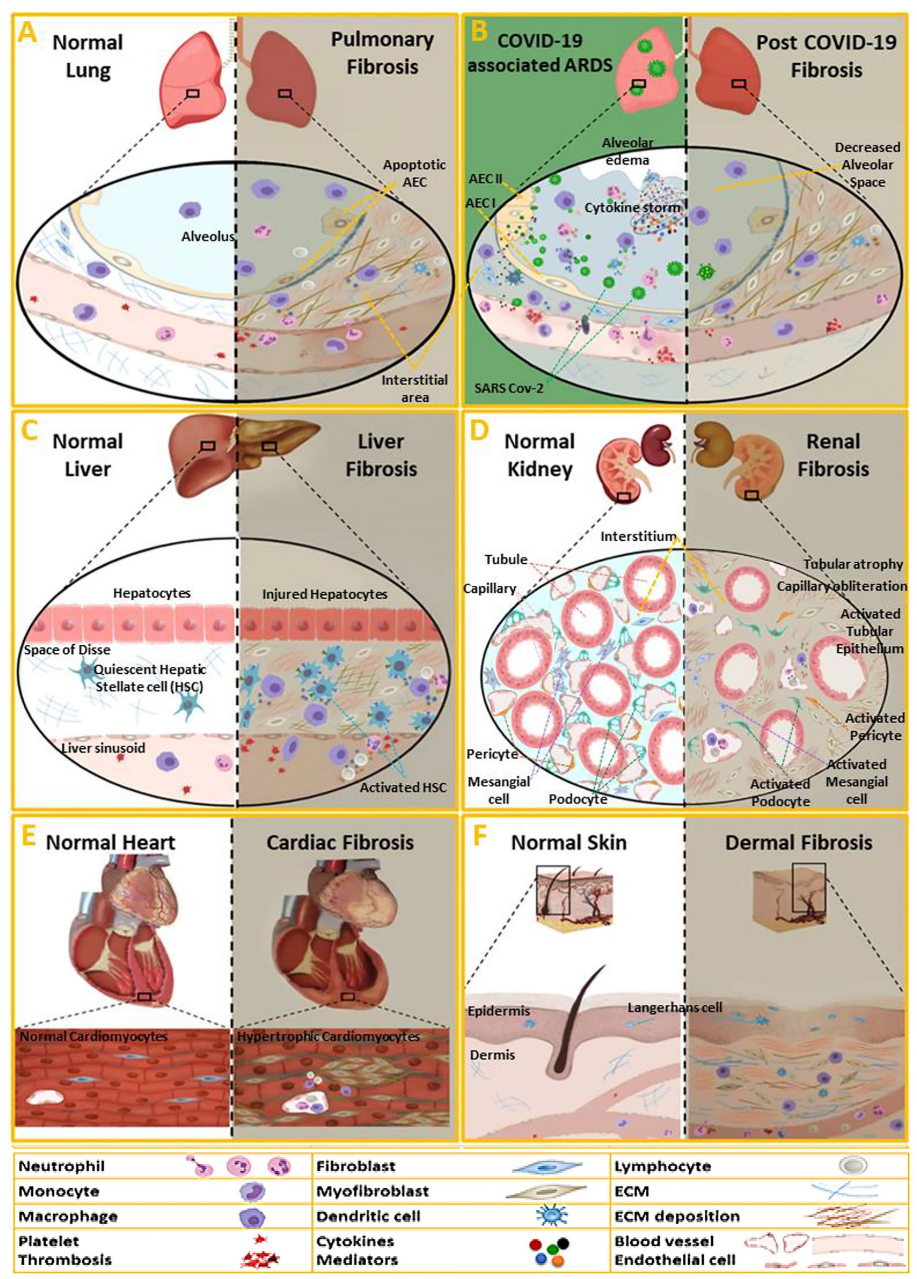
The main contributing factors in the pathogenesis of fibrotic lesions in various organs. (**A**, **C**, **D**, **E** and **F**) Schematic illustrations of the normal and fibrotic states of various organs, including the heart, liver, kidney, and skin. (**B**) Schematic illustration of the acute respiratory distress syndrome (ARDS) and pulmonary fibrosis associated with the SARS-CoV-2 infection. The fibrotic events and involved cells are outlined. For more details, please refer to the text

Pathogenesis of IPF is complex, but the chronic inflammatory process and persistently epithelial-dependent fibroblast-activation, and overproduction of collagen within the lung tissue are central events [176]. Repetitive injury and dysfunction of alveolar epithelial cells (AECs), as an initial step in IPF, form the inflammatory early stage [177]. The alveolar-capillary membrane disrupts and alveolar epithelial and endothelial cells undergo apoptosis. Apoptotic cells recruit a variety of inflammatory cells and lead to lung tissue regeneration. Damaged epithelial cells and recruited inflammatory cells to produce TGF-β, PDGF, CTGF, fibroblast growth factor (FGF), vascular endothelial growth factor (VEGF), and other pro-fibrotic mediators. These mediators promote epithelial cell apoptosis, EMT, the proliferation of fibroblasts, and the differentiation and activation of collagen-producing myofibroblasts (Fig. 2A) [178]. Other pro-fibrotic growth factors such as insulin-like growth factor-1 (IGF-1), and cytokines such as IL-4, which favor eliciting a type 2 immune response, also increase IPF [179], along with a reduction in anti-fibrotic factors such as IFN-γ inducible protein-10 (IP-10) [180]. AEC2s not only release a large amount of pro-fibrotic mediators but also lose the ability to produce anti-fibrotic mediators, such as prostaglandin E2 (PGE2) [25]. As the most potent pro-fibrotic mediator, TGF-β promotes these functions in IPF through various signaling pathways, which mainly include the Smad, MAPK, PI3K, ERK, and Wnt/β-catenin [181].

Moreover, immune dysregulation plays a critical role in the development of IPF [182]. Different cell types from both innate and adaptive immune systems, often with conflicting findings, have been related to IPF pathogenesis. The disturbed balance in wound healing processes implicating IPF is supposed to be mainly orchestrated by alveolar macrophages [183]. The number of macrophages increases in the lungs of IPF patients and they are involved in IPF pathogenesis in either a pro- (previously known as M1) or anti-fibrotic (previously known as M2) manner. Macrophages, through interplay with T cells, can disturb the balance of the Th1/Th2 immune response in the lung [184]. Macrophages further contribute to ECM shaping by secretion of MMPs and tissue inhibitor of metalloproteinase (TIMPs), among other secreted factors, such as collagen and fibronectin [183, 185]. The imbalance between MMPs and their inhibitors (TIMPs), through the upregulation of TIMPs within the lung parenchyma and reducing the MMP-induced degradation of the ECM, is involved in fibrogenesis [186]. Ultimately, these pathological changes and destruction of alveolar architecture cause a progressive decline in lung function, leading to end-stage respiratory failure and serious comorbidities, such as lung cancer [187].

Despite this, there is presently no curative for IPF. Although, Corticosteroids, anti-inflammatory agents, and immune-suppressive drugs, are used experimentally to treat IPF for many years, have no beneficial effects on survival or disease progression [188–190], and occasionally worsen disease outcomes during clinical IPF trials [191]. Two new FDA-approved anti-fibrotic agents, pirfenidone and nintedanib, retarded disease progression in phase III studies, however, did not improve survival and quality of life [192]. Meanwhile, they exhibited marked adverse effects [193]. Lung transplantation, with many inconveniences and restrictions, is the only available curative intervention [194]. Hence, more effective, safe, and convenient treatment strategies remained a critical need. Stem cells offer a new strategy for IPF therapy owing to immunomodulatory, anti-inflammatory and anti-fibrotic characteristics.

Transplanted MSCs enter damaged lungs and contribute to the improvement of pulmonary fibrosis through direct intercellular interactions, or in a paracrine manner by secretome (secreted soluble bioactive products and extracellular vesicles). Thereby, MSCs mediate their tissue-repairing and immunomodulatory effects [50]. For tissue repairing, MSCs suppress apoptosis of the alveolar epithelial cells and endothelial cells, and promote re-epithelialization and angiogenesis, via secretion of anti-apoptotic mediators and growth factors, such as keratinocyte growth factor (KGF), epidermal growth factor (EGF), hepatocyte growth factor (HGF), and angiopoietin-1 (Ang1) [195]. Some stress signals within involved lung tissue, such as hypoxia, further can stimulate MSCs to enhance these therapeutic effects [196]. Hypoxic preconditioning of MSCs prior to transplantation showed to result in improved protection from pulmonary fibrosis [197], at least partially, via increased production of VEGF and HGF [198]. However, it has been shown that MSCs downregulate VEGF, along with nitric oxide metabolites and pro-inflammatory cytokines [199].

For immunomodulation, MSCs produce a series of anti-inflammatory mediators, including IL-10, IL-4, IL-1RA, soluble TNFR1, IFN-γ, PGE2, and IDO-1 [50]. MSCs also exert immunomodulatory activities via secretion of stanniocalcin-1 (STC1) and − 2 (STC2) under stress conditions, which are involved in anti-oxidative and anti-inflammation properties promoting the beneficial effects of MSCs in IPF. It has been shown that MSCs enhance STC1 secretion via PI3/AKT/mTORC1 pathway, and decreased oxidative stress and endoplasmic reticulum (ER)-stress, thereby downregulating TGF-β1 in AECs and macrophages [200, 201]. Furthermore, MSCs via secretome or cellular contact interact with innate and adaptive immune cells to suppress the T-cell proliferation, induce the regulatory lymphocytes, reduce the B cell activation and proliferation, apoptosis of the CD8^+^ T cells, inhibit the NK cell cytotoxicity, alteration of DC maturation [202], and downregulate the pro-fibrotic lung macrophages [203]. Moreover, MSCs directly exert an anti-scarring effect by collagen degradation and inhibiting lung remodeling via regulation of the MMPs/TIMPs balance [204].

iPSCs and secretomes obtained from iPSCs, contribute to alveolar epithelial repair, suppress inflammatory responses, and improve IPF. Transplanted iPSCs trapped in damaged lungs secrete HGF that reduces AEC apoptosis and improve epithelial growth [170]. Another factor that mediates anti-pulmonary fibrosis effects of iPSC is IP-10, which plays a role in the modulation of lymphocyte and neutrophil infiltrations, and inhibition of fibroblast accumulation [24]. iPSCs regulate the macrophages’ phenotype and their secretions toward lung repair and regeneration [172, 173].

iPSCs also, can downregulate the pro-fibrotic growth factors IGF-1,2 [205], and repress several inflammatory mediators during pulmonary fibrosis, including TNF-α, IL-1β, IL-6, inducible nitric oxide synthase (iNOS) and nitric oxide (NO), but also PGE2. They also suppress the EMT process in lung tissues through upregulation of epithelial marker E-cadherin and downregulation of mesenchymal markers such as fibronectin, vimentin, and α-SMA. Importantly, iPSCs promote these effects by mitigating of TGF-β1/Smad2/3 pathway. In addition, iPSCs modulate MMPs/TIMPs ratios, preventing collagen deposition in pulmonary tissues [156, 171].

#### COVID-19

SARS-CoV-2 is a newly worldwide-distributed coronavirus, which mainly involves the respiratory system besides other affecting organs. Although the infection with this virus mostly is self-limited, some patients manifest acute respiratory distress syndrome (ARDS), lung fibrosis, and subsequent multi-organ failure and death [206]. These pathologic manifestations have been indicated to be caused by cytokine storm, which is created by uncontrolled levels of pro-inflammatory cytokines released in the lungs, due to the immune hyper-reaction provoked by the SARS-CoV-2 virus [207, 208]. The excessive pro-inflammatory cytokines and chemokines, including IL-1β, IL-2, IL-6, IL-7, IL-8, IL-33, TNFα, granulocyte colony-stimulating factor (GSCF), IP-10, monocyte chemoattractant protein 1 (MCP1), macrophage inflammatory protein 1(MIP1), and TGF-β, extremely secreted by a massive inflammatory cell infiltration, and recruit to the further immune cells, promoting a pro-inflammatory feedback loop [209]. These inflammatory events damage the architecture of the lungs, which subsequently undergo repair and remodeling via fibroproliferation. Thereby, can lead to pulmonary fibrosis as the COVID-19 outcome (Fig. 2B) [210].

MSCs have recently emerged potential approach to hamper the excessive inflammatory responses and cytokine storm in the lungs, as well as to regenerate and restore the functional lung tissue in COVID-19 patients. The mechanisms of MSCs that researchers relied on which for repairing, counteracting fibrosis, and improvement of lung function in COVID-19 respiratory disease, are similar to those employed by MSCs in IPF [88, 211]. MSCs could remediate immune-pathological cytokine storm through the secretion of anti-inflammatory mediators and increase Treg cells, Th22 cells, and M2 macrophage phenotype [210]. Although it needs strong evidence to be confirmed, one of the possible pathways underlying this anti-inflammatory function of MSCs can be involved in the suppression of myeloid differentiation factor 88 (MyD88) adaptor protein. MyD88 is utilized by TLR7 on macrophages upon recognizing single-stranded RNA from viruses such as SARS-CoV-2. This can lead to activating nuclear factor-kB (NF-kB), which is a transcription factor inducing the expression of pro-inflammatory factors mediating lung damage in COVID-19 [212]. MSCs were shown to mitigate the MyD88 signaling axis, which is suggested to play a key role in the inflammation and pathogenesis of pulmonary fibrosis [91].

### Liver fibrosis

Liver fibrosis developed as an intrinsic response to chronic persistent liver damage caused by multiple noxious factors such as viral infection, drugs, alcoholism, non-alcoholic fatty liver disorder, and autoimmune diseases, which trigger the cycles of hepatocytes apoptosis, inflammation, and repetitive wound healing process, leading to ECM deposition and fibrous scar formation [6]. EMT is an essential contributor to liver fibrosis. Epithelial-derived mesenchymal cells that are generated during EMT, undergo a subsequent mesenchymal-to-epithelial transition (MET) to convert into hepatocytes or cholangiocytes for repairing damaged liver. This repair becomes fibrogenic in the chronically injured liver, where EMT activity surpasses MET [213, 214]. Hepatic stellate cells (HSCs) play a crucial role in this process, upon the activation, and subsequent proliferation and transformation into myofibroblasts with increased expression of TGF-β, PDGF, CTGF, VEGF, TIMP1, α-SMA, and type I collagen [215]. The activation of kupffer cells, liver resident macrophages, is considered an important contributing factor in the activation of HSCs, via the secreting the various mediators, including oxidants, cytokines (TGF-β, TNF-α, IL-1, and IL-6) and proteinases (Fig. 2C) [216]. . Several signaling pathways were known to be involved in HSC activation, such as TGF-β/Smad, Wnt/β-catenin, Ras/ERK, and Notch [217]. These fibrotic events can progress to liver cirrhosis, referred to as end-stage liver fibrosis with no effective treatment that requires orthotopic liver transplantation as the only therapeutic option [218].

Transplanted MSCs contribute to liver regeneration directly through differentiation into hepatocyte-like cells, and indirectly by releasing factors for immune regulation. Anti-liver fibrosis effects of MSCs are exerted majorly in a paracrine manner. MSCs stimulate liver cell proliferation and inhibit apoptosis of hepatocytes and sinusoidal endothelial cells (SECs), via anti-apoptotic factors such as HGF and IGF-1 [219]. MSCs promote liver regeneration by expression of VEGF and MMP9 [220], and downregulation of TIMP1 in the injured liver [221]. MSCs enhance hepatocyte proliferation by the production of PGE2 via YAP and mTOR signaling [222]. In addition, MSC-derived PGE2 can promote the anti-inflammatory M2 phenotype of liver macrophages through STAT6 mTOR signaling. MSC-derived PGE2 decreases macrophage-produced inflammatory cytokines via inhibiting the TGF-β-activated kinase 1 (TAK1) signaling and NLRP3 inflammasome activation [223]. . Furthermore, MSCs suppress the secretion of inflammatory cytokine by T cells, B cells, NK cells, and DCs [224–226], and participate in converting the CD4^+^ T cells into anti-inflammatory Tregs and Th2 cells [227]. MSCs further promote inflammatory resolution by reducing the infiltration of neutrophils and macrophages [220, 228]. MSCs can also exert anti-liver fibrosis activity by alleviating the process of EMT [82], as well as promoting the process of MET [229]. MSCs inhibit the activation of HSCs and kupffer cells. MSCs downregulate TGF-β1 receptor expression through secreting milk fat globule-EGF factor 8 (MFGE8), which binds to αβ integrin on hepatic stellate cells (HSCs) and thereby inhibits the activation of HSCs [230]. MSCs also inhibit the HSC activation via suppressing the Wnt/β-catenin pathway [231]. MSCs can repress the pro-fibrotic TGF-β/Smad signaling pathway; thereby reducing hepatic collagen deposition and α-SMA expression [81, 232].

Human ESCs and particularly iPSCs, provide a great promise as a supply for parenchymal and non-parenchymal liver cells [233]. iPSC-derived hepatocytes enhance liver regeneration and reduce liver fibrosis [234, 235]. EVs produced by iPSCs can modulate HSC activation and expression of TIMP-1, mediated by anti-fibrotic miRNAs [174]. Moreover, Exosomes from iPSC-derived MSCs activate the sphingosine kinase and sphingosine1-phosphate pathway in hepatocytes and promote cell proliferation, thereby alleviating liver fibrosis [236].

### Cardiac fibrosis

Cardiac fibrosis is developed following most types of cardiac injury. These injuries result in; (a) extensive cardiomyocyte death such as myocardial infarction (MI), stimulating healing response and replacement of dead cells with fibroblasts that lead to deposition of ECM proteins and myocardial scar formation; (b) non-extensive cardiomyocyte loss such as pressure or volume overload, hypertrophic cardiomyopathy and cardiomyopathy induced by diabetes, obesity and brief ischemic events without completed infarction. In which, interstitial and perivascular deposition of collagen develops as an adaptive response to keep the pressure-generating ability of the heart in the dysfunctional myocardial segments that can progress into the replacement of fibrous tissue (Fig. 2E) [237]. Generally, excessive ECM deposition in the myocardium, interrupts myocyte–myocyte interactions and leads to ventricular wall stiffness with diastolic and systolic dysfunction, and causes electric instability promoting arrhythmia that may result in irreversible heart failure and death [238, 239]. As in fibrosis of other organs, initial inflammatory reaction with upregulation of cytokines and pro-fibrotic mediators, subsequent activation of fibrogenic pathways, and activation of proteases in response to persistent cardiac injury are prominent components in the pathogenesis of myocardial remodeling [237]. During the inflammatory response, macrophages and other inflammatory cells secrete various cytokines and pro-fibrotic mediators including TGF-β, TNF-α, CTGF, PDGF, fibroblast growth factor (FGF), and monocyte chemoattractant protein (MCP)-1 to provoke the differentiation and proliferation of myofibroblasts [240]. In the fibrotic heart, myofibroblasts originate from resident fibroblasts, epithelial cells (through EMT), and circulating fibrocytes, monocytes, and progenitor cells derived from bone marrow [241].

Although the principal aim of stem cell-based therapy in cardiac disease and heart failure is to replenish the cardiac tissue, new findings demonstrated that pluripotent stem cells, consisting iPSCs and ESCs, and/or their derivatives, mediate the restoration of heart function by their paracrine activity. ESCs reduce adverse cardiac remodeling via triggering myocardial regeneration, attenuating collagen deposition, secreting anti-apoptotic proteins cystatin C, osteopontin, and clusterin, and anti-fibrotic factors such as TIMP-1 [120]. ESCs can also promote differentiation of resident cardiac stem cells, and thereby endogenous cardiac regeneration, via realizing HGF and IGF-1 [242]. Numerous studies demonstrated the beneficial effect of iPSCs and cardiomyocyte-like cells generated from iPSCs in alleviating adverse remodeling and improving cardiac function [243], probably via direct or paracrine mechanisms that remain to be elucidated. iPSCs and their conditioned media inhibit apoptosis and reduce interstitial and vascular fibrosis in the heart [244]. Dual stem cell therapy by applying iPSC-derived cardiomyocytes and MSCs restore heart function and enhance vessel formation post-MI. In this manner, intra-myocardial infusion of iPSC-derived cardiomyocytes leads to improvement of cardiac function by engraftment with the host myocardium, and epicardial implanted MSC patches concurrently promote vascular regeneration via consistent secretion of angiogenic factors. MSCs contribute to cardiac repair through their paracrine factors with pleiotropic effects, including pro-angiogenesis, anti-inflammation, anti-fibrosis, and CM maturation [245]. Transplanted MSCs produce HGF, VEGF and fibroblast growth factor 2 (FGF2) that stimulate cell survival, angiogenesis and neovascularization [246]. HGF is a potent anti-fibrotic released by MSCs transplanted into the area around MI and inhibits miR-155-mediated profibrotic signaling, thereby reducing left ventricular remodeling and preventing fibrosis [247]. MSC-derived PGE2 inhibits TGF-β expression, collagen accumulation, and myocardial fibrosis [248]. MSCs also produce MMP 2, 9, and 14 to inhibit fibroblast activation and ECM deposition, thus improving cardiac fibrosis [249]. Moreover, MSCs modified to overexpress IGF-1, can reduce the myofiber area in the cardiac muscle [250], and miR133-overexpressing-MSCs attenuate fibrosis triggered by MI via suppressing Snail 1, which is considered the master regulator of EMT [251].

### Renal fibrosis

Regardless of underlying etiology, renal fibrosis is a common pathological process of various progressive kidney injuries and is regarded as a therapeutic target for CKD [252]. Fibrogenesis can occur in the different kidney compartments inclusive of glomerulus, tubules, or vessels, which are referred to as glomerulosclerosis, tubulointerstitial fibrosis, and arteriosclerosis, respectively [252]. However, they share the key fibrotic mechanisms including the loss of epithelial cells and capillary bed, infiltration of inflammatory cells, activation of fibroblasts, accumulation of activated myofibroblasts, and ECM [253]. Eventually, ECM accumulation continues unchecked during chronic injury that occurs in CKD, which may lead to end-stage kidney failure that needs lifelong dialysis or kidney transplantation [254].

Renal fibrosis is mostly preceded by inflammation occurred secondary to excessive kidney epithelial cell injury, which is induced by various causes including ischemia, toxins, advanced glycation products, and proteinuria originating from different diseases such as diabetes, hypertension, and glomerulonephritis. However, under several conditions including viral or bacterial infections, autoimmune disease, and after transplantation, epithelial injury develops following inflammatory responses. Following epithelial injury and expression of pro-inflammatory cytokines, the influx of macrophages, T-cells, and mast cells is increased [255]. Infiltrated inflammatory cells release molecules that damage tissues such as ROS, and promote the secretion of pro-fibrotic cytokines and growth factors [256, 257]. Moreover, paracrine factors produced by epithelial cells such as TGF-β, CTGF, PDGF, FGF, TNF, angiotensin II, and aldosterone, trigger the transformation of myofibroblasts to produce a large amount of ECM components [255]. In the tubulointerstitium, activated myofibroblasts predominantly derivate from resident fibroblasts and pericytes [258]. Myofibroblastic activation of the mesangial cells is important in ECM production [259]. Moreover, trans-differentiation of the podocytes undergoing EMT causes more ECM deposition (Fig. 2D) [260, 261]. Several signaling pathways have been strongly correlated with mediating these fibrotic events in CKD and renal fibrosis; In which, TGF- β/Smad signaling is a central pathway considering the extensive cross-talks with other pro-fibrotic pathways [262]. Nuclear factor-kappaB (NF-κB) mediates the overproduction of cytokines participating in the fibrotic process [263, 264], and the induction and maintenance of EMT [265]. Sustained damage in CKD induces excessive activation of the Wnt and Notch pathways in the epithelial cells, which in turn interact synergistically with Hedgehog signaling to mediate renal fibrosis. Wnt and Notch overexpression inhibits the terminal differentiation of renal epithelial cells and up-regulated Wnt and Hh expression promotes fibroblast proliferation and myofibroblastic transformation in the kidney [255]. Besides, PI3K/AKT/mTOR, mitogen-activated protein kinase (MAPK), and RHO/Rho coil kinase (ROCK) signaling pathways are important in the regulation of EMT and progression of renal fibrosis [266–270].

Stem cell therapy retards the progression of renal fibrosis. MSCs constitute the vast majority of SCs used for renal fibrosis treatment so far. MSCs and their conditioned medium prohibit renal fibrosis by diminishing EMT and reducing ECM deposition in the kidney [72, 271, 272]. MSCs or their EVs attenuate oxidative damage and apoptosis, as well as improve renal tubular cell proliferation and capillary density. These functions were observed along with increasing HGF, IL-10, heme oxygenase-1 (HO-1) and reducing ROS, NADPH oxidase 2 (NOX2), BAX, and CTGF [273–276]. Transplanted MSCs can improve hypoxic tubulointerstitial conditions and decrease HIF-1α, thereby, upregulating VEGF expression [277, 278]. These cells promote a pro-angiogenic microenvironment with an increased level of VEGF, Ang I, and decreased Flt1 expression, allowing the injured renal capillary bed to repair. VEGF signaling enhances the endothelial cell survival and proliferation as well as the formation of new vessels; and is negatively regulated by Flt1, which is a receptor for decoy VEGF. Whereas Ang I contributes to the capillary structure strengthening and maintenance of vascular stability [279]. However, some reports are indicating administrated MSCs did not change VEGF levels during renal fibrosis improvement [280, 281]. Anti-renal fibrotic effects of MSCs are also associated with the downregulation of pro-fibrotic mediators and pro-inflammatory cytokines such as TGF-β1, PDGFR-β, TNF-α, IL-6, IL-1β, MIP-2, and MCP-1 and alleviation of renal neutrophil and macrophage infiltration [282, 283]. Besides, their EVs decrease the number of M1 macrophages and increase M2 macrophages in the inflamed kidney [284].

Transplanted MSCs ameliorate renal fibrosis by hampering the fibrotic signaling pathways, and mainly inhibit the activation of TGF-β1/Smads, NF-κB, and ERK (as the main subsets of MAPK signaling), PI3K/AKT. Furthermore, MSC-derived EVs suppress the RhoA/ROCK pathway via MFGE8 [276, 285–287]. In addition, MSCs can decrease the MMP-9 expression during tubulointerstitial fibrosis and increase the TIMP-1/MMP-9 ratio, in part, by suppressing STAT3 activation [288]. Although a limited number of studies examined the impact of ESCs on renal fibrosis, transplanted ESCs were able to hinder the progression of CKD, and reduce glomerulosclerosis and tubular injury [289]. The underlying mechanism appears to be involved in the decreased inflammatory infiltrate, tubular apoptosis, and renal oxidative stress via upregulated the antioxidant enzyme HO-1 [290]. iPSCs are also capable of reducing macrophage infiltration, tubular atrophy, interstitial fibrosis, and glomerulosclerosis. Furthermore, iPSCs upregulate the expression of the VEGF gene [291, 292]. The anti-renal fibrosis activity of iPSCs appears to be majorly developed through a paracrine effect [291]. iPSCs-derived secretome exerts antioxidant, anti-inflammatory, and anti-apoptotic effects on renal damage induced by ischemia-reperfusion [293]. iPSCs-derived conditioned medium is capable of reducing cell death, ROS production, and inflammatory cytokine responses, as well as protecting functional mitochondria, thereby improving renal function [294, 295]. Furthermore, iPSC-derived MSCs display comparable effects in the improvement of renal function, including decreasing cell apoptosis and promoting vascularization with adult MSCs [296].

### Dermal fibrosis

Fibrotic skin disorders, either those associated with dysregulated cutaneous wound healing that occurred in response to dermal injuries such as hypertrophic scars and keloids, or those associated with metabolic and immunological disorders, such as scleroderma, share several pathological features, comprised fibroblast over-proliferation, ECM over-production, and loss of skin elasticity (Fig. 2F) [297, 298].

Cutaneous wound healing normally is a transient process, in which, most wounds take no longer than 2 to 3 weeks to heal [299]. However, pathological scarring can be induced by devastating insults, such as deep burns, infected wounds, and extensive trauma, following dysregulated wound healing [300, 301]. Keloids recognized as benign fibrotic tumors, which are raised scars, tend to be larger than the original wound site, spontaneously regress extremely seldom, and often recur after surgical excision. While hypertrophic scars grow within the confines of the primary wound border, frequently regress spontaneously, and rarely recur after incision [302, 303]. Besides the cosmetic issues, discomfort, and psychological stress; pathological scars can also be associated with dysfunction, infection, itching, and pain, hence seriously impairing the quality of life [304, 305]. The reticular layer of hypertrophic scars and keloids is characterized by infiltration of inflammatory cells, increased frequency of fibroblasts, newly formed blood vessels, and collagen deposits, particularly types I and III. In addition, pro-inflammatory mediators, such as TNF-α, IL-1, and IL-6 are upregulated in keloid tissues. Moreover, keloid fibroblasts (KFs) display faster proliferation, more ECM production, and more invasiveness compared with normal fibroblasts [306]. They also express elevated levels of biologically active isoforms of TGF-β ligands and their receptors, therefore, KFs exert a unique sensitivity to TGF-β stimulation [307]. Besides TGF-β, IGF-1 and VEGF contribute to several aspects of abnormal scarring, including ECM deposition, cell proliferation, inflammation, immunoreaction, and angiogenesis [308, 309].

Scleroderma is considered the prototype of fibrosing connective tissue diseases of the skin and exists in two types; systemic sclerosis (SSc), the life-threatening disease with further involvement of internal organs such as lung and kidneys in addition to the skin; and localized scleroderma, in which the fibrotic changes of internal organs are absent and life prognosis is not compromised [297]. Apart from the functional defects of involved organs in systemic form, both demonstrate cutaneous symptoms that are frequently accompanied by pain, physical appearance deformity, and psychological stress. The main pathogenic constituents of scleroderma are (i) microangiopathy due to structural damage of small vessels, (ii) autoimmunity along with the production of auto-antibodies and activation of T cells, (iii) skin fibrosis results from an excessive ECM deposition [310, 311]. Increased production of collagen I and III fibers following the inflammatory fibrotic response forms a compact wax-like intensely fibrotic matrix in the dermis. Further, hyaluronan markedly accumulates within the epidermis and dermis, in particular around blood vessels [297]. In addition to the increased production, degradation of ECM is also inhibited by autoantibodies blocking MMP-1 and MMP-3, which are found in scleroderma patients [312–314]. Simultaneously, up-regulated expression of TIMPs, such as TIMP-1, might further contribute to increasing the extent of fibrosis into late-stage disease [297, 315]. The predominant T-helper (Th) lymphocytic infiltrate in the skin lesions, and elevated level of related cytokines, including TGF-β, CTGF, PDGF, TNF-α, IL-1, IL-2, IL-3, IL-4, IL-6, IL-13 and IL-17 in patients, are involved in the histopathologic features of skin fibrosis in the SSc and localized scleroderma [311, 316, 317]. Moreover, skin fibroblasts from patients with SSc exert resistance to Fas-mediated apoptosis due to TGF-β-induced Akt activation [318]. The other scleroderma-like conditions, such as scleroderma, lichen sclerosis, eosinophilic fasciitis (Shulman’s disease), and graft-versus-host disease (GVHD), share a dysregulated ECM turnover resulting in excessive cutaneous collagen accumulation by activated fibroblasts. However, the underlying mechanisms, cutaneous manifestations and systemic implications are different. These fibrosing skin disorders are often incurable, and effective treatments remain to be established [297].

The therapeutic potential of MSCs in hypertrophic scars is attributed to the higher expression of important anti-fibrotic mediators, such as TGF-β3 and HGF [319]. TGF-β3 enables to antagonization of the pro-fibrotic function of TGF-β1 [320]. MSCs are also involved in the elevated expression of MMP-2 and a higher MMP-2/TIMP-2 ratio, which reflect the remodeling activity to reverse fibrosis. These effects are associated with reduced dermis thickness and skin collagen content in the humanized skin graft model in nude mice [319]. The other mechanism by which MSCs prevent hypertrophic scar formation is the secretion of TNF-alpha-stimulated gene/protein 6 (TSG-6) under apoptosis, and inflammatory regulation [321]. The anti-scarring effect of MSCs is partly mediated by the suppression of the p38/MAPK signaling pathway [106]. MSCs can inhibit the proliferation, migration, and protein expression of collagen I and III, through suppression of the TGF-β1/Smad2/3/7) pathway in hypertrophic scar fibroblasts (HSFs) and KFs [322, 323]. Additionally, the anti-fibrotic effect on these fibroblasts is mediated by downregulation of the pro-fibrotic mediators, such as CTGF, PAI-1, TGF-β1 and 2, as well as upregulation of the anti-fibrotic mediators, such as TGF-β3 and decorin in HSFs and KFs through a paracrine manner [324].

Furthermore, MSCs inhibit inflammatory cell accumulation, angiogenesis, and collagen deposition, thereby, keloid development via paracrine secretions. The therapeutic mechanism is mediated partially by Ectodysplasin-A2 (EDA-A2), Insulin-like growth factor binding protein-related protein-1 (IGFBP-rp1) /IGFBP-7, and TSP-1 [325]. The inhibitory mechanism of MSCs on dermal fibroblast growth and induce apoptosis in the keloids, is involved in the inhibiting proliferation of KFs and promoting their apoptosis by regulating the arachidonic acid-derived cyclooxygenase-2 (COX-2)/PGE2 pathway, through a paracrine manner [326]. In this way, MSCs further, inhibit KF-related bioactivities, including proliferation, migration, cellular invasion, and ECM production, through the blockade of TGF-β/Smad and MAPK/ERK signaling pathways [327].

Contrary to the effects observed with AD-MSCs and BM-MSCs, WJ-MSCs appear to promote keloid phenotype through a paracrine signaling mechanism. WJ-MSC-CM can enhance the expression of the pro-fibrotic gene, PAI-1, and TGF-β2, downregulated the expression of the anti-fibrotic gene, TGF-β3, increased the level of pro-fibrotic proteins, IL-6, IL-8, TGF-β1 and 2, in KFs. The secretome of WJ-MSCs promotes the proliferation of KFs, with no significant change in their apoptosis rate or migration ability [328]. Besides, BM-MSCs have been reported to enhance the fibrotic behavior of deep dermal fibroblasts through paracrine signaling [329].

The therapeutic mechanism of MSCs in the systemic form of scleroderma includes the induction of apoptosis in activated T cells via activation of the Fas/Fas ligand pathway, leading to ameliorating autoimmune phenotypes and reducing hypodermal thickness [330, 331]. MSCs ameliorate BLM-induced scleroderma by preventing the infiltration of CD4^+^ and CD8^+^ T cells and macrophages into the dermis. They not only downregulate the expression of collagen and pro-fibrotic cytokines, such as IL-6 and IL-13, in the skin but also reduce the frequency of pro-fibrotic cytokine-producing CD4^+^ T cells and effector B cells in the spleen [332]. In addition, MSCs or their exosomes transfer miR-151-5p into recipient cells and attenuate autoimmune and dermal phenotypes of fibrosis, accompanied by an improvement of osteopenia in *Tsk*/^+^ mice, via regulating the IL-4 receptor alpha (IL4Rα)/mTOR pathway [333]. MSC administration ameliorates BLM-induced lung and skin fibrosis and accelerates wound healing, associated with downregulated expression of pro-fibrotic miR-199 and increase of corresponding protein expression of its target, caveolin-1 (CAV-1). Furthermore, they inhibit BLM-induced overexpression of α_v_-integrin and TNF-α in lung and skin wounds, as well as suppression of AKT activation [334].

In addition to MSCs, iPSCCM can be effective in preventing hypertrophic scar formation. iPSCCM reduces collagen and αSMA in dermal fibroblasts activated with TGF-β1. Moreover, activation and contractibility of fibroblast, as well as recruitment and adhesion of inflammatory cells are hampered by iPSCCM [335]. In scleroderma, iPSC-derived ECs reduce collagen content, the number of cells, and skin fibrosis, in addition to participating in damaged vessel recovery [336]. However, additional research is needed to elucidate the mechanism of the suppressive effect on dermal fibrosis by iPSCs.

## Resident stem cells for organ-specific fibrosis therapy

In addition to various exogenous stem cell sources explored for their anti-fibrotic potential, resident stem cells present within affected organs themselves hold promise as a future therapeutic strategy. These organ-specific stem cells are thought to be involved in tissue maintenance, repair, and regeneration throughout life. Their inherent localization within the target organ and potential immunologic compatibility offer potential advantages compared to stem cells derived from other sources.

### Resident stem cells in different organs

#### Lungs:

The lung epithelium harbors resident stem cells, including basal epithelial stem cells and club cells, crucial for maintaining the alveolar surface. Additionally, mesenchymal stromal cells reside within the lung interstitium and contribute to tissue repair [337]. Studies are underway to investigate the therapeutic potential of these cells for pulmonary fibrosis by promoting regeneration and modulating the immune response; Concurrently, instances where these cells may inadvertently contribute to lung fibrosis are also under investigation, with the aim of discovering innovative methods to steer their activity towards beneficial outcomes.“ [338–340].

#### Liver:

Hepatic progenitor cells reside in the canals of Hering and are believed to be responsible for liver regeneration after injury [341]. Liver-derived human mesenchymal stem cells (LHMSCs) have been isolated from the liver, LHMSCs share characteristics with other MSCs but possess unique features as they may produce higher levels of beneficial factors compared to other MSCs, potentially making them more effective. Also, being liver-derived, they might have a natural affinity for liver tissue and function [342]. LHMSCs could be a promising therapeutic approach for various liver diseases due to their regenerative and immunomodulatory properties. Research is ongoing to explore their therapeutic application in liver fibrosis models, with promising results in reducing fibrosis and promoting hepatocyte proliferation [341–343].

#### Kidneys:

The kidneys harbor renal progenitor cells within the nephron structure. These cells play a role in kidney repair after injury, and their potential for therapeutic use in kidney fibrosis is being explored [344, 345]. However, compared to lungs and liver, research on resident kidney stem cells for fibrosis therapy remains at an earlier stage. Also, they have the potential to contribute to kidney fibrosis themselves; as in investigating the role of resident mesenchymal stem-like cells (MSLCs) in kidney fibrosis caused by ureteral obstruction (UUO) in mice, it was observed that MSLCs from the obstructed kidney increased their expression of genes associated with fibrosis (collagen, inflammatory factors, TGF-beta) [346].

#### Heart:

The heart harbors a population of cardiac progenitor cells (CPCs) residing within the myocardium and epicardium [347]. These cells are thought to contribute to cardiac repair after injury, although their exact role and regenerative potential are still under investigation. Preclinical studies suggest CPCs may hold promise for treating heart failure and myocardial infarction, potentially through mechanisms involving paracrine signaling and immunomodulation [348]. In exploring the potential of CPCs as a treatment for cardiac fibrosis using a sophisticated 3D model, it was observed that CPCs co-cultured with human cardiac fibroblasts reduced the fibrotic response, suggesting an anti-fibrotic effect [349]. Further research is needed to determine their efficacy and safety for treating cardiac fibrosis [350].

#### Skin:

The epidermis, the outermost layer of the skin, contains epidermal stem cells responsible for lifelong renewal of the skin surface [351]. These stem cells also contribute to wound healing after injury [352, 353]. While not directly studied in the context of fibrosis yet, their regenerative potential suggests they might be a future avenue for exploring therapies targeting skin fibrosis.

### Current efforts to leverage resident stem cells

While significant challenges remain regarding isolation, expansion, and complete understanding of their functions, current efforts to utilize resident stem cells for treating fibrosis include:

- **Optimizing isolation and expansion techniques**: Researchers are developing methods to efficiently isolate resident stem cells from target organs with minimal damage and expand them in culture for therapeutic use. This involves identifying specific markers that distinguish these cells and developing culture conditions that support their self-renewal and differentiation potential [354–356].
- **Gene editing and cell engineering**: Gene editing techniques like CRISPR-Cas9 are being explored to modify resident stem cells and enhance their therapeutic potential. This could involve introducing genes that promote tissue regeneration or immunomodulation, or correcting genetic abnormalities that might contribute to fibrosis [357–359].
- **Delivery methods and scaffolding techniques**: Researchers are investigating methods to safely deliver resident stem cells to the target organ within the body. This might involve using biocompatible scaffolds or hydrogels to support cell engraftment and survival at the site of injury or fibrosis [360–363].
- **Preclinical studies in animal models**: Studies are ongoing in animal models of fibrosis to evaluate the efficacy and safety of resident stem cell therapies. These studies assess the ability of these cells to reduce fibrosis, improve organ function, and promote tissue regeneration [350, 364].

### Challenges and considerations

Despite the exciting potential of resident stem cells, significant challenges remain due to a Limited Understanding of the underlying mechanism by which these cells could affect fibrotic processes since our knowledge regarding the differentiation capacity and regenerative potential of these cells is still evolving. Also, efficient methods for isolating and expanding resident stem cells for therapeutic use require further development. Furthermore, Safety Considerations must be taken into account because manipulating resident stem cell populations might carry unforeseen risks, requiring careful evaluation. Overall, resident stem cells within various organs represent a promising approach for organ-specific fibrosis therapy. While challenges exist regarding their isolation, expansion, and complete understanding of their functions, ongoing research holds promise for the development of novel therapeutic strategies to combat fibrosis and promote tissue regeneration. It should be noted that some studies pointed to the profibrotic effects of these cells which necessitates a thorough insight into the signaling pathways contributing to fibrotic processes; this facilitates harnessing the anti-fibrotic capacities of these cells by insightful manipulation of their physiological properties toward combating fibrosis with various techniques such as genetic engineering methods.

## Molecular signaling pathways in anti-fibrosis: unveiling the cellular crosstalk and potential of stem cell therapies

Fibrosis development is a complex orchestration of cellular processes driven by intricate molecular signaling pathways. Understanding these pathways is crucial for developing effective therapeutic strategies to combat fibrosis in various organs. This section explores some key signaling molecules and their roles in fibrosis, along with current efforts to target them therapeutically using antifibrotic agents and the emerging potential of stem cell therapies:

### Transforming growth factor-β (TGF-β): a master fibrogenic regulator

TGF-β is a potent cytokine that plays a central role in fibrosis initiation and progression [15]. It activates Smad proteins, which translocate to the nucleus and regulate the expression of genes involved in extracellular matrix (ECM) production, myofibroblast differentiation, and pro-inflammatory responses. Some therapeutic Strategies include using antifibrotic agents such as antibodies and small molecule inhibitors; Inhibiting TGF-β signaling using specific antibodies or small molecule inhibitors holds promise as a therapeutic strategy. Some examples include Fresolimumab (monoclonal antibody against TGF-β1) which is undergoing clinical trials for various fibrotic conditions [365–367]. The small molecules pirfenidone (blocks Smad signaling) as well as [368](multi-targeted tyrosine kinase inhibitor) which are approved for the treatment of idiopathic pulmonary fibrosis [369]. Among the stem cells discussed so far, mesenchymal stem cells (MSCs) have shown promise in pre-clinical studies to counteract TGF-β signaling [364, 370]. MSCs can secrete immunomodulatory factors that suppress TGF-β activity and promote tissue repair [371, 372]. Additionally, gene editing approaches could be explored to engineer MSCs with enhanced anti-TGF-β capabilities. Although there are studies reporting TGF-b production by the MSCs which limits the safe application of these cells for antifibrotic approaches [371]. Besides, iPSCs have also shown promises in treating mouse models of IPF by downregulating the TGF-β and the downstream pathways [156].

### Mitogen-activated protein kinase (MAPK) pathways: a network of fibrogenic signaling

The MAPK family, including ERK, JNK, and p38, are activated by various stimuli and contribute to fibrosis development [373–375]. These pathways regulate cell proliferation, survival, migration, and production of pro-fibrotic mediators. Targeting specific MAPK pathways using inhibitors could be explored for the treatment of organ fibrosis [373]. Some examples include: Ulixertinib a selective ERK1/2 inhibitor [376]; JNK-specific inhibitors which are still in pre-clinical stages of development but might show promise for targeting fibrosis [376]; and Losmapimod (p38 MAPK inhibitor) which also could be used for the treatment of fibrosis [377]. Besides these approaches, MSCs have also demonstrated the ability to modulate MAPK signaling pathways [378, 379]. Further research is needed to fully understand the complex interactions between MSCs and MAPK signaling in fibrosis. It should be noted that due to the plasticity and heterogeneity of MSCs, they could also enhance the activity of this pathway [380].

### Mammalian target of rapamycin (mTOR) pathway: a regulator of cell growth and metabolism

The mTOR pathway plays a crucial role in cell growth, proliferation, and metabolism [381]. Dysregulation of mTOR signaling has been implicated in fibrosis development by promoting myofibroblast activation and ECM production [382]. mTOR inhibitors are being investigated for their potential to prevent or reverse fibrosis [383]. Some examples including Everolimus (mTORC1 inhibitor) and Sirolimus (mTORC1/2 inhibitor) are approved for other conditions but are being explored for their antifibrotic potential as well [384–387]. On the other hand, Pre-clinical studies suggest that MSCs can suppress mTOR activity, potentially contributing to their anti-fibrotic effects [387]. However, the exact mechanisms by which MSCs modulate mTOR signaling in fibrosis are still under investigation.

### Wnt/β-catenin signaling: a double-edged sword in fibrosis

The Wnt/β-catenin pathway has complex and context-dependent roles in fibrosis [388]. Both canonical and non-canonical Wnt/β-catenin signaling can promote fibrosis. Modulating specific components of the Wnt pathway offers potential for targeted therapeutic strategies [389, 390]. However, this area of research is still evolving, and specific inhibitors are not yet widely used in clinical trials for fibrosis. Emerging evidence suggests that certain stem cell populations, such as induced pluripotent stem cells (iPSCs), can modulate Wnt signaling [391]. Furthermore, iPSCs have the potential to differentiate into various cell types within the affected organ, potentially influencing the Wnt signaling landscape in multiple ways.

### Organ-specific signaling crosstalk: tailoring therapies

The specific signaling pathways contributing to fibrosis can vary depending on the affected organ [392]. Understanding these organ-specific differences is crucial for developing targeted therapies. For example, integrin signaling plays a significant role in liver fibrosis, while Notch signaling might be more relevant in kidney fibrosis [255, 393]. Research continues to unravel the intricacies of molecular signaling pathways involved in fibrosis. By elucidating the complex crosstalk between these pathways and their influence on different cell types, researchers can develop more effective therapeutic strategies tailored to specific organs and fibrosis subtypes [394]. This will lead the way for personalized medicine approaches to combat fibrosis and improve patient outcomes.

## Future directions and perspectives for stem cell-based therapy for fibrotic diseases are

- To optimize the delivery methods, doses, and timing of stem cell administration to achieve the best anti-fibrotic outcomes and minimize the potential adverse effects, such as tumorigenicity, immunogenicity, and infection.
- To identify the optimal sources, types, and subpopulations of stem cells that have the highest anti-fibrotic potency and specificity for different organs and diseases.
- To elucidate the molecular mechanisms and pathways by which stem cells modulate the fibrotic process, such as by secreting anti-inflammatory and anti-fibrotic factors, transferring mitochondria or exosomes, or directly differentiating into functional cells.
- To develop novel strategies to enhance the anti-fibrotic effects of stem cells, such as by genetic engineering, preconditioning, or combination with other therapies, such as drugs, biomaterials, or gene therapy.
- To establish standardized and validated protocols and criteria for the isolation, expansion, characterization, and quality control of stem cells for clinical use.
- To conduct more rigorous and large-scale clinical trials to evaluate the safety and efficacy of stem cell-based therapy for fibrotic diseases, as well as to monitor the long-term outcomes and potential complications.

## Conclusion

Despite the encouraging advances in the understanding of the pathophysiology of organ fibrosis, effective treatment to stop the progression towards organ failure remained an urgent need. In recent years, stem cells have shed great light on anti-fibrotic therapy. A growing body of evidence supports the remarkable impact of stem cell-based therapy on various fibrotic mechanisms and repair processes. Although, the results of the in-vitro and in-vivo studies mentioned above are promising, several challenges and issues currently remain to be addressed before such therapeutic strategy can be safely translated to clinical practice for patients with fibrotic diseases: (i) well-defining and standardizing the optimal dose, route, time and course of stem cell administration; (ii) optimization and refinement of isolation, reprogramming (in the case of iPSC), purification, characterization, cultivation, propagation, differentiation and pretreatment protocols; (iii) Considering safety issues, including immunogenic risks, clear assessing oncogenic transformation risk, prospective elimination of tumorigenic cells through using intrinsic cell properties, such as surface antigens, to minimize the tumorigenesis potential of transplanted stem cells, determining the mechanisms of homing and long term safety of utilizing stem cells; (iv) Recognizing the exact anti-fibrotic mechanisms of stem cells particularly iPSCs, as unrestricted sources of pluripotent stem cells, in organ fibrosis. Given that most investigations in this field considered MSCs for stem cell therapy, further endeavors are required to recognize mechanisms and pathways by which, iPSCs conduct the suppressive effect to stop fibrogenesis in various organs. While stem cell therapy for fibrosis shows promise, there’s a gap between lab investigations and large-scale clinical applications. MSCs are currently leading the way due to their safety, feasibility, and encouraging results in ongoing clinical trials. Drawing definitive conclusions about safety and effectiveness requires more comprehensive clinical trial data. However, the ongoing research with MSCs suggests this approach has the potential to move from the lab towards clinical application in the coming years (Table 2). Collectively, taking these issues into account allows for enhancing the efficacy and avoiding the adverse effects of stem cell-based therapy, which improves survival and reduces the mortality rates in millions of patients suffering from organ fibrosis.

**Table 2:** Summary of the clinical studies regarding the utilization of stem cells in the treatment of fibrotic disorders

NCT Number	Study Title	Study URL	Study Status	Conditions	Interventions	Collaborators	Phases
NCT01220492	Umbilical Cord Mesenchymal Stem Cells for Patients With Liver Cirrhosis	https://clinicaltrials.gov/study/NCT01220492	COMPLETED	Liver Cirrhosis	DRUG: conventional plus MSC treatment|DRUG: conventional plus placebo treatment		PHASE1|PHASE2
NCT01013194	Human Fetal Liver Cell Transplantation in Chronic Liver Failure	https://clinicaltrials.gov/study/NCT01013194	COMPLETED	Liver Cirrhosis	OTHER: Human Fetal Liver Cell Transplantation	University of Pittsburgh	PHASE1|PHASE2
NCT05948982	Safety of Umbilical Cord Mesenchymal Stem Cells (UC-MSC) in Patients With Decompensated Hepatitis B Cirrhosis	https://clinicaltrials.gov/study/NCT05948982	NOT_YET_RECRUITING	Decompensated Liver Cirrhosis	BIOLOGICAL: Human Umbilical Cord Mesenchymal Stem Cells		PHASE1|PHASE2
NCT01120925	Autologous Bone Marrow Derived Stem Cells in Decompensate Cirrhotic Patients	https://clinicaltrials.gov/study/NCT01120925	COMPLETED	Liver Cirrhosis	BIOLOGICAL: MNC|BIOLOGICAL: CD133|BIOLOGICAL: Control	University of Tehran	PHASE1|PHASE2
NCT01981330	Pilot Study of Stem Cell Treatment of Patients With Vocal Fold Scarring	https://clinicaltrials.gov/study/NCT01981330	COMPLETED	Improved Healing of Scarred Vocal Folds|Improved Vocal Fold Status|Improved Vocal Fold Function	BIOLOGICAL: aMSC|BIOLOGICAL: aMSC + hyaluronan gel	The Swedish Research Council|Laryngfonden|Karolinska Institutet	PHASE1
NCT02594839	Safety and Efficacy of Allogeneic Mesenchymal Stem Cells in Patients With Rapidly Progressive Interstitial Lung Disease	https://clinicaltrials.gov/study/NCT02594839	COMPLETED	Idiopathic Interstitial Pneumonia|Interstitial Lung Disease|Idiopathic Pulmonary Fibrosis	DRUG: Bone marrow mesenchymal stem cells|DRUG: Placebo		PHASE1|PHASE2
NCT06230822	Safety, Tolerability and Efficacy of VUM02 Injection in Treatment of Idiopathic Pulmonary Fibrosis (IPF)	https://clinicaltrials.gov/study/NCT06230822	RECRUITING	Idiopathic Pulmonary Fibrosis	DRUG: VUM02 Injection		PHASE1
NCT05871463	Effect of Mesenchymal Stem Cells-derived Exosomes in Decompensated Liver Cirrhosis	https://clinicaltrials.gov/study/NCT05871463	RECRUITING	Decompensated Liver Cirrhosis	BIOLOGICAL: MSC-derived exosomes		PHASE2
NCT04243681	Combination of Autologous MSC and HSC Infusion in Patients With Decompensated Cirrhosis	https://clinicaltrials.gov/study/NCT04243681	COMPLETED	Cirrhosis, Liver	COMBINATION_PRODUCT: CD 34 and MSC infusion|DRUG: Standard of care for Cirrhosis management		PHASE4
NCT05191381	Immune Modulation by Exosomes in COVID-19	https://clinicaltrials.gov/study/NCT05191381	RECRUITING	COVID-19|Critical Illness|Hypercytokinemia|Lung Fibrosis	BIOLOGICAL: Application of exosomes in a whole blood assay		
NCT05106972	Umbilical Cord Mesenchymal Stem Cell Transplantation for Decompensated Hepatitis B Cirrhosis	https://clinicaltrials.gov/study/NCT05106972	RECRUITING	Liver Cirrhosis	DRUG: UC-MSC infusion		NA
NCT05224960	Human Umbilical Cord-derived Mesenchymal Stem Cells for Decompensated Cirrhosis (MSC-DLC-2)	https://clinicaltrials.gov/study/NCT05224960	NOT_YET_RECRUITING	Decompensated Cirrhosis	BIOLOGICAL: UC-MSCs|BIOLOGICAL: Placebo(solution without UC-MSCs)	Chinese PLA General Hospital|Shanghai Changzheng Hospital|LanZhou University|Renmin Hospital of Wuhan University|Jin Yin-tan Hospital|Hainan Hospital of Chinese PLA General Hospital|Vcanbio Cell and Gene Engineering Corp., Ltd.	PHASE2
NCT01919827	Study of Autologous Mesenchymal Stem Cells to Treat Idiopathic Pulmonary Fibrosis	https://clinicaltrials.gov/study/NCT01919827	COMPLETED	Idiopathic Pulmonary Fibrosis	BIOLOGICAL: Endobronchial infusion of adult mesenchymal stem cells|BIOLOGICAL: Autologous mesenchymal stem cells derived from bone marrow		PHASE1
NCT01432080	Steroids, Azithromycin, Montelukast, and Symbicort (SAMS) for Viral Respiratory Tract Infection Post Allotransplant	https://clinicaltrials.gov/study/NCT01432080	TERMINATED	Respiratory Tract Infections|Bronchiolitis Obliterans|Cryptogenic Organizing Pneumonia|Lung Diseases, Interstitial	DRUG: Prednisone|DRUG: Azithromycin|DRUG: Montelukast|DRUG: Symbicort	The Canadian Blood and Marrow Transplant Group	PHASE2
NCT00476060	Mesenchymal Stem Cell Transplantation in Decompensated Cirrhosis	https://clinicaltrials.gov/study/NCT00476060	COMPLETED	Cirrhosis	PROCEDURE: Autologous mesenchymal stem cell transplantation		PHASE2
NCT02772289	Perinatal Tissue Mesenchyme Stem Cells in the Treatment for Caesarean Section Scars	https://clinicaltrials.gov/study/NCT02772289	COMPLETED	Cicatrix	BIOLOGICAL: Mesenchyme Stem Cells low-dose group|BIOLOGICAL: Mesenchyme Stem Cells high-dose group|BIOLOGICAL: Placebo		PHASE2
NCT05507762	Study of Human Umbilical Cord Mesenchymal Stem Cell in Patients With Cirrhosis Due to Hepatitis B (Compensation Stage)	https://clinicaltrials.gov/study/NCT05507762	RECRUITING	Cirrhosis Due to Hepatitis B	BIOLOGICAL: UC-MSCs|BIOLOGICAL: Saline solution	Vcanbio Cell and Gene Engineering Corp., Ltd.|Wuhan Optics Valley Zhongyuan Pharmaceutical Co., Ltd., Hubei, China	PHASE1|PHASE2
NCT05227846	Human Umbilical Cord-derived Mesenchymal Stem Cells for Decompensated Cirrhosis (MSC-DLC-1)	https://clinicaltrials.gov/study/NCT05227846	RECRUITING	Decompensated Cirrhosis	BIOLOGICAL: Human Umbilical Cord-derived Mesenchymal Stem Cells	Vcanbio Cell and Gene Engineering Corp., Ltd.	PHASE1
NCT01385644	A Study to Evaluate the Potential Role of Mesenchymal Stem Cells in the Treatment of Idiopathic Pulmonary Fibrosis	https://clinicaltrials.gov/study/NCT01385644	COMPLETED	Idiopathic Pulmonary Fibrosis	OTHER: Placental MSC	Mater Medical Research Institute	PHASE1
NCT04262167	Human Autologous Lung Stem Cell Transplant for Idiopathic Pulmonary Fibrosis	https://clinicaltrials.gov/study/NCT04262167	RECRUITING	Idiopathic Pulmonary Fibrosis	BIOLOGICAL: Lung Spheroid Stem Cells 100 million|BIOLOGICAL: Lung Spheroid Stem Cells 200 million		PHASE1
NCT02297867	Clinical Trial Study About Human Adipose-Derived Stem Cells in the Liver Cirrhosis	https://clinicaltrials.gov/study/NCT02297867	COMPLETED	Liver Cirrhosis	DRUG: ADSCs		PHASE1
NCT05887804	Comparison of Keloid Volume and Symptoms Reduction Between Intralesional Umbilical-Cord Mesenchymal Stem Cells, Its Conditioned Medium, and Triamcinolone Acetonide Injection as Keloid Therapy: A Randomised Controlled Trial	https://clinicaltrials.gov/study/NCT05887804	COMPLETED	Keloid|Stem Cell	BIOLOGICAL: umbilical cord-derived mesenchymal stem cells (UC-MSC)|BIOLOGICAL: umbilical cord-derived mesenchymal stem cells conditioned medium (UC-CM)|DRUG: Triamcinolone Acetonide (TA)	Indonesia University	PHASE4
NCT05080465	Long Term Follow up Mesenchymal Stem Cell Therapy for Patients Virus-related Liver Cirrhosis	https://clinicaltrials.gov/study/NCT05080465	ACTIVE_NOT_RECRUITING	Liver Cirrhosis	BIOLOGICAL: Autologous BM MSC		PHASE3
NCT03500731	Lung and Bone Marrow Transplantation for Lung and Bone Marrow Failure	https://clinicaltrials.gov/study/NCT03500731	RECRUITING	Idiopathic Pulmonary Fibrosis|Emphysema or COPD	BIOLOGICAL: CD3/CD19 negative hematopoietic stem cells|DRUG: Rituximab|DRUG: Alemtuzumab|DRUG: Fludarabine|DRUG: Thiotepa|DRUG: G-CSF|DRUG: Hydroxyurea		PHASE1|PHASE2
NCT00147043	Adult Stem Cell Therapy in Liver Insufficiency	https://clinicaltrials.gov/study/NCT00147043	COMPLETED	Liver Cirrhosis	PROCEDURE: Leukapheresis|PROCEDURE: Infusion of stem cells via image guided scan		NA
NCT05468502	Phase I/IIa Clinical Trial of Human Umbilical Cord Mesenchymal Stem Cell Injection in the Treatment of Idiopathic Pulmonary Fibrosis (IPF)	https://clinicaltrials.gov/study/NCT05468502	RECRUITING	Idiopathic Pulmonary Fibrosis	DRUG: Human umbilical cord mesenchymal stem cell injection		PHASE1
NCT02745184	Clinical Efficacy and Safety of Autologous Lung Stem Cell Transplantation in Patients With Idiopathic Pulmonary Fibrosis	https://clinicaltrials.gov/study/NCT02745184	COMPLETED	Idiopathic Pulmonary Fibrosis	BIOLOGICAL: Lung stem cells	Regend Therapeutics	PHASE1|PHASE2
NCT03887208	Therapy of Scars and Cutis Laxa With Autologous Adipose Derived Mesenchymal Stem Cells	https://clinicaltrials.gov/study/NCT03887208	COMPLETED	Skin|Scar|Cutis Laxa|Keloid|Cicatrix	PROCEDURE: Laser therapy|BIOLOGICAL: Autologous ADSC injection|PROCEDURE: Normal saline injection	Timeless Chirurgia Plastyczna-Janusz Jaworowski|Melitus sp. z o.o.|Polish Stem Cells Bank S.A.	PHASE1|PHASE2
NCT01875081	REVIVE(Randomized Exploratory Clinical Trial to Evaluate the Safety and Effectiveness of Stem Cell Product in Alcoholic Liver Cirrhosis Patient)	https://clinicaltrials.gov/study/NCT01875081	COMPLETED	Alcoholic Liver Cirrhosis	BIOLOGICAL: Livercellgram		PHASE2
NCT01342250	Human Umbilical Cord Mesenchymal Stem Cells Transplantation for Patients With Decompensated Liver Cirrhosis	https://clinicaltrials.gov/study/NCT01342250	COMPLETED	Liver Cirrhosis	BIOLOGICAL: conventional therapy plus low dose hUC-MSCs treatment|BIOLOGICAL: conventional therapy plus medium dose hUC-MSCs treatment|BIOLOGICAL: conventional therapy plus high dose hUC-MSCs treatment	No.85 Hospital, Changning, Shanghai, China	PHASE1|PHASE2
NCT02801890	Evaluation of Autologous Adipose Derived Mesenchymal Stromal Cells (AD-MSC) Transplantation in Ultra Filtration Failure (UFF)	https://clinicaltrials.gov/study/NCT02801890	COMPLETED	Ultra Filtration Failure	BIOLOGICAL: Intravenous injection		PHASE1|PHASE2
NCT03460795	Safety and Efficacy Study of Co-transfering of Mesenchymal Stem Cell and Regulatory T Cells in Treating End-stage Liver Disease	https://clinicaltrials.gov/study/NCT03460795	NOT_YET_RECRUITING	Liver Cirrhosis	BIOLOGICAL: MSC and Tregs		PHASE1|PHASE2
NCT05984303	Human Umbilical Cord-derived Mesenchymal Stem Cells for Decompensated Cirrhosis (MSC-DLC-1b)	https://clinicaltrials.gov/study/NCT05984303	NOT_YET_RECRUITING	Decompensated Cirrhosis	BIOLOGICAL: Human Umbilical Cord-derived Mesenchymal Stem Cells	Wuhan Optics Valley Zhongyuan Pharmaceutical Co., Ltd., Hubei, China	PHASE1
NCT05939817	The Effect of Intralesian Injection of Umbilical Cord Mesenchymal Stem Cells, Its Conditioned Medium, and Triamcinolone Acetonide on Type 1:3 Collagen Ratio and Interleukin-10 Levels in Keloid: A Randomised Controlled Trial	https://clinicaltrials.gov/study/NCT05939817	COMPLETED	Keloid|Stem Cell	BIOLOGICAL: umbilical cord-derived mesenchymal stem cells (UC-MSC)|BIOLOGICAL: umbilical cord-derived mesenchymal stem cells conditioned medium (UC-CM)|DRUG: Triamcinolone Acetonide (TA)	Indonesia University	PHASE4
NCT00713934	Autologous Bone Marrow Stem Cells in Cirrhosis Patients	https://clinicaltrials.gov/study/NCT00713934	COMPLETED	Stem Cell Transplantation|Cirrhosis	BIOLOGICAL: CD133|BIOLOGICAL: BM-MNC	Small Business Developing Center|Shiraz University of Medical Sciences	PHASE1|PHASE2
NCT05016817	Safety of Cultured Allogeneic Adult Umbilical Cord Derived Mesenchymal Stem Cell Intravenous Infusion for IPF	https://clinicaltrials.gov/study/NCT05016817	RECRUITING	Idiopathic Pulmonary Fibrosis	BIOLOGICAL: AlloRx		PHASE1
NCT00420134	Improvement of Liver Function in Liver Cirrhosis Patients After Autologous Mesenchymal Stem Cell Injection: a Phase I-II Clinical Trial	https://clinicaltrials.gov/study/NCT00420134	COMPLETED	Liver Failure|Cirrhosis	PROCEDURE: injection of progenitor of hepatocyte drived from Mesenchymal stem cell	Tarbiat Modarres University	PHASE1|PHASE2
NCT04326036	Use of cSVF Via IV Deployment for Residual Lung Damage After Symptomatic COVID-19 Infection	https://clinicaltrials.gov/study/NCT04326036	ENROLLING_BY_INVITATION	Pulmonary Alveolar Proteinosis|COPD|Idiopathic Pulmonary Fibrosis|Viral Pneumonia|Coronavirus Infection|Interstitial Lung Disease	PROCEDURE: Microcannula Harvest Adipose Derived tissue stromal vascular fraction (tSVF)|DEVICE: Centricyte 1000|PROCEDURE: IV Deployment Of cSVF In Sterile Normal Saline IV Solution|DRUG: Liberase Enzyme (Roche)|DRUG: Sterile Normal Saline for Intravenous Use	Robert W. Alexander, MD	EARLY_PHASE1
NCT01454336	Transplantation of Autologous Mesenchymal Stem Cell in Decompensate Cirrhotic Patients With Pioglitazone	https://clinicaltrials.gov/study/NCT01454336	COMPLETED	Liver Fibrosis	BIOLOGICAL: Cell injection		PHASE1
NCT05925036	Novel Cellular Therapy for the Treatment of Pain Associated With Chronic Pancreatitis	https://clinicaltrials.gov/study/NCT05925036	RECRUITING	Chronic Pancreatitis|Chronic Pain	DRUG: Mesenchymal stem cells|OTHER: Placebo		PHASE1
NCT05698134	Rotational Thromboelastometry (ROTEMâ„¢) Guided Transfusion for Elective Procedures in Patients With Cirrhosis (REduCe): An Open Label Randomized Controlled Trial.	https://clinicaltrials.gov/study/NCT05698134	RECRUITING	Cirrhosis, Liver	DIAGNOSTIC_TEST: ROTEM|OTHER: Standard of care		NA
NCT04088058	A Phase II Open-label Single-arm Study to Evaluate the Efficacy and Safety of ADSCs in Subjects With Liver Cirrhosis	https://clinicaltrials.gov/study/NCT04088058	RECRUITING	Liver Cirrhosis	DRUG: GXHPC1		PHASE2
NCT03254758	A Study of ADR-001 in Patients With Liver Cirrhosis	https://clinicaltrials.gov/study/NCT03254758	COMPLETED	Decompensated Liver Cirrhosis	BIOLOGICAL: Mesenchymal stem cell		PHASE1|PHASE2
NCT03724617	Clinical Study of Umbilical Cord Mesenchymal Stem Cells Combined With Collagen Scaffold in the Treatment of Thin Endometrium	https://clinicaltrials.gov/study/NCT03724617	COMPLETED	Thin Endometrium|Intrauterine Adhesion	DRUG: umbilical cord mesenchymal stem cells combined with collagen scaffold		NA
NCT05155657	Study of Decompensated Alcoholic Cirrhosis Treatment by Stem Cells	https://clinicaltrials.gov/study/NCT05155657	RECRUITING	Alcoholic Cirrhosis	BIOLOGICAL: Conventional therapy plus low dose UCMSCs treatment|BIOLOGICAL: Conventional therapy plus medium dose UCMSCs treatment|BIOLOGICAL: Conventional therapy plus high dose UCMSCs treatment		PHASE1
NCT04357600	Umbilical Cord Mesenchymal Stem Cell for Liver Cirrhosis Patient Caused by Hepatitis B	https://clinicaltrials.gov/study/NCT04357600	RECRUITING	Liver Cirrhoses	BIOLOGICAL: Allogeneic Umbilical Cord Mesenchymal Stem Cell		PHASE1|PHASE2
NCT02013700	Allogeneic Human Cells (hMSC)in Patients With Idiopathic Pulmonary Fibrosis Via Intravenous Delivery (AETHER)	https://clinicaltrials.gov/study/NCT02013700	TERMINATED	Idiopathic Pulmonary Fibrosis (IPF)	BIOLOGICAL: Allogeneic Adult Human Mesenchymal Stem Cells (hMSCs)|BIOLOGICAL: matched placebo	The Lester And Sue Smith Foundation|The Emmes Company, LLC	PHASE1
NCT03472742	An Follow-Up Study of Liver Cirrhosis	https://clinicaltrials.gov/study/NCT03472742	COMPLETED	Decompensated Liver Cirrhosis	DRUG: Mesenchymal Stem Cell		
NCT02941705	Regression of Fibrosis & Reversal of Diastolic Dysfunction in HFpEF Patients Treated With Allogeneic CDCs	https://clinicaltrials.gov/study/NCT02941705	COMPLETED	Congestive Heart Failure|Heart Failure, Diastolic	BIOLOGICAL: Allogeneic Derived Cells|BIOLOGICAL: Placebo/Control Arm	Medical University of South Carolina	PHASE2
NCT02277145	A Study on Radiation-induced Pulmonary Fibrosis Treated With Clinical Grade Umbilical Cord Mesenchymal Stem Cells	https://clinicaltrials.gov/study/NCT02277145	COMPLETED	Post-radiotherapy Pulmonary Fibrosis	BIOLOGICAL: clinical grade umbilical cord mesenchymal stem cells	Southwest Hospital, China	PHASE1
NCT03826433	hUC Mesenchymal Stem Cells (19#iSCLifeÂ®-LC) in the Treatment of Decompensated Hepatitis b Cirrhosishepatitis b Cirrhosis	https://clinicaltrials.gov/study/NCT03826433	RECRUITING	Hepatitis B	BIOLOGICAL: Peripheral iv		PHASE1
NCT03333187	Ruxolitinib vs. Allogeneic SCT for Patients With Myelofibrosis According to Donor Availability	https://clinicaltrials.gov/study/NCT03333187	ACTIVE_NOT_RECRUITING	Bone Marrow Fibrosis	PROCEDURE: Allogeneic stem cell transplantation|DRUG: Ruxolitinib continuous therapy	Novartis|Clinical Trial Center North (CTC North GmbH & Co. KG)	PHASE2
NCT05984628	Umbilical Cord Stem Cells for Skin Grafts in Donor Site Wounds	https://clinicaltrials.gov/study/NCT05984628	RECRUITING	Skin Wound|Scar, Hypertrophic	PROCEDURE: human umbilical cord mesenchymal stem cells|PROCEDURE: blank solvent		NA
NCT03352297	Nanofat in Post Burn Scars on the Face	https://clinicaltrials.gov/study/NCT03352297	COMPLETED	Burn Scar	BIOLOGICAL: Unfiltered Nanofat Graft		NA
NCT00382278	Safety Study of Autologous Stem Cell in Liver Cirrhosis	https://clinicaltrials.gov/study/NCT00382278	TERMINATED	Liver Cirrhosis	PROCEDURE: Autologous bone marrow-derived mononuclear cells infusion	Financiadora de Estudos e Projetos	PHASE1|PHASE2
NCT04553159	Autologous Adipose Derived Stem Cells Transplantation in the Treatment of Keloids.	https://clinicaltrials.gov/study/NCT04553159	COMPLETED	Keloid|Autologus Adipose Derived Stem Cells|Feasibility|Safety	BIOLOGICAL: Autologous adipose derived stem cells		PHASE2
NCT01591200	Dose Finding Study to Assess Safety and Efficacy of Stem Cells in Liver Cirrhosis	https://clinicaltrials.gov/study/NCT01591200	COMPLETED	Alcoholic Liver Cirrhosis	BIOLOGICAL: Allogeneic Mesenchymal Stem Cells|BIOLOGICAL: Allogeneic Mesenchymal Stem Cells|BIOLOGICAL: Allogeneic Mesenchymal Stem Cells		PHASE2
NCT04689152	Clinical Trial to Evaluate the Efficacy and Safety of Cellgram-LC Administration in Patients With Alcoholic Cirrhosis	https://clinicaltrials.gov/study/NCT04689152	RECRUITING	Alcoholic Cirrhosis	BIOLOGICAL: Cellgram-LC		PHASE3
NCT05442437	Clinical Study of hUC-MSCs Treating Decompensated Liver Cirrhosis With HBV	https://clinicaltrials.gov/study/NCT05442437	COMPLETED	HBV	BIOLOGICAL: hUCMSCs		EARLY_PHASE1
NCT01333228	Evaluate Safety and Efficacy of Autologous Bone Marrow-derived Endothelial Progenitor Cells in Advanced Liver Cirrhosis	https://clinicaltrials.gov/study/NCT01333228	COMPLETED	Liver Cirrhosis	OTHER: Autologous bone marrow-derived endothelial progenitor cells	Foundation Ramon Areces|Instituto de Salud Carlos III	PHASE1|PHASE2

## Data Availability

Not applicable.

## Abbreviations

IL-10: interleukin-10
IL-4: interleukin-4
IL-1ra: IL-1 receptor antagonist
IFN-γ: interferon-γ
PGE2: prostaglandin E2
IDO-1: indolamine 2,3-dioxygenase-1
Ang-1: angiogenin-1
HGF: hepatocyte growth factor
KGF: keratinocyte growth factor
